# Computational approach to modeling microbiome landscapes associated with chronic human disease progression

**DOI:** 10.1371/journal.pcbi.1010373

**Published:** 2022-08-04

**Authors:** Lu Li, Jiho Sohn, Robert J. Genco, Jean Wactawski-Wende, Steve Goodison, Patricia I. Diaz, Yijun Sun

**Affiliations:** 1 Department of Computer Science and Engineering, University at Buffalo, The State University of New York, Buffalo, New York, United States of America; 2 Department of Medicine, University at Buffalo, The State University of New York, Buffalo, New York, United States of America; 3 Department of Oral Biology, University at Buffalo, The State University of New York, Buffalo, New York, United States of America; 4 UB Microbiome Center, University at Buffalo, The State University of New York, Buffalo, New York, United States of America; 5 Department of Epidemiology and Environmental Health, University at Buffalo, The State University of New York, Buffalo, New York, United States of America; 6 Department of Quantitative Health Sciences, Mayo Clinic, Jacksonville, Florida, United States of America; 7 Department of Microbiology and Immunology, University at Buffalo, The State University of New York, Buffalo, New York, United States of America; University of Trento, ITALY

## Abstract

A microbial community is a dynamic system undergoing constant change in response to internal and external stimuli. These changes can have significant implications for human health. However, due to the difficulty in obtaining longitudinal samples, the study of the dynamic relationship between the microbiome and human health remains a challenge. Here, we introduce a novel computational strategy that uses massive cross-sectional sample data to model microbiome landscapes associated with chronic disease development. The strategy is based on the rationale that each static sample provides a snapshot of the disease process, and if the number of samples is sufficiently large, the footprints of individual samples populate progression trajectories, which enables us to recover disease progression paths along a microbiome landscape by using computational approaches. To demonstrate the validity of the proposed strategy, we developed a bioinformatics pipeline and applied it to a gut microbiome dataset available from a Crohn’s disease study. Our analysis resulted in one of the first working models of microbial progression for Crohn’s disease. We performed a series of interrogations to validate the constructed model. Our analysis suggested that the model recapitulated the longitudinal progression of microbial dysbiosis during the known clinical trajectory of Crohn’s disease. By overcoming restrictions associated with complex longitudinal sampling, the proposed strategy can provide valuable insights into the role of the microbiome in the pathogenesis of chronic disease and facilitate the shift of the field from descriptive research to mechanistic studies.

## Author summary

The delineation of system dynamics of a microbial community can provide a wealth of insights into the roles of the microbiome in the pathogenesis of chronic disease. However, due to the difficulty in obtaining longitudinal samples, most existing microbiome studies have been cross-sectional and largely descriptive. Here, we present a novel computational strategy that leverages massive static sample data to model microbiome landscapes associated with chronic disease development. To demonstrate the validity of the proposed strategy, we applied it to a gut microbiome dataset available from a Crohn’s disease study and constructed one of the first microbial progression models of the disease. We performed a series of interrogations on the constructed model. Our analysis suggested that the constructed model recapitulated the longitudinal progression of microbial dysbiosis during the known clinical trajectory of Crohn’s disease. By overcoming the sampling restrictions inherent to slowly progressive diseases, our approach is potentially widely applicable in many different studies across body sites, diseases, and other conditions.

This is a *PLOS Computational Biology* Methods paper.

## Introduction

The human microbiome—trillions of microbes residing in and on human bodies—plays an essential role in many important physiological processes. Studies that are part of the Human Microbiome Project and others have significantly expanded our knowledge of the human microbiota and its implications for human health [1–5]. However, most microbiome studies performed to date have been cross-sectional, using single time-point data to examine the potential role of the microbiome in human health. While cross-sectional studies are a logical first step, these analyses are largely descriptive and provide little information about microbial community dynamics with respect to disease development. A possible way to elucidate system dynamics in this context is to assemble time-series data through repeated sampling of the same cohort of subjects across a defined disease process. This could provide a wealth of insights into pathogenesis that is unattainable through a static experimental design. However, due to economical and logistical constraints, time-course studies have generally been limited by the number of samples examined and the time period followed, and consequently data collected may only cover a partial picture of microbial dynamics [6–9]. This is particularly true when studying chronic diseases (i.e., Crohn’s disease or periodontitis), the development of which can take decades. Consequently, it has been difficult to study microbial community dynamics and their possible contribution to the initiation and progression of human chronic diseases.

As cost-effective DNA sequencing technology continues to advance, large-scale epidemiological studies are providing access to data from many thousands of microbiome samples. This provides us with a unique opportunity to develop an analytical strategy that uses massive cross-sectional data, instead of time-course data, to study microbial community dynamics in disease. The strategy is based on the rationale that each static sample provides a snapshot of the disease process, and if the number of samples is sufficiently large, the footprints of individual samples populate progression trajectories, which in turn enables the recovery of microbial community dynamics by using computational approaches. To demonstrate the validity of the proposed strategy, we developed a bioinformatics pipeline and applied it to a gut microbiome dataset available from a Crohn’s disease (CD) study [7]. CD is a chronic inflammatory disease characterized by discontinuous lesions that can affect the entire gastrointestinal tract. It tends to start in the teens and twenties, though it can occur at any age [10], and as there are no curative interventions currently available [11], it is considered a lifelong illness. At diagnosis, most patients present with a clinical inflammatory behavior, but stricturing or penetrating complications develop as the disease progresses [12, 13]. The extent of CD lesions also changes overtime, initially involving either the ileum or the colon and later progressing to the ileocolonic region [12]. Previous metagenomics studies suggested that CD results from aberrant immune responses to the intestinal microbiota [14, 15], but details of how microbiome shifts initiate or promote disease development, progression and symptom exacerbation are lacking. Our analysis using the developed bioinformatics pipeline revealed a double bifurcating model of microbial alterations that occur during disease development. The constructed model was validated by aligning it with clinical and molecular traits. The analysis suggested that the identified microbiome trajectories reflected changes in CD behavior, location and severity associated with disease progression. To further demonstrate the utility of the model, we projected the samples onto the identified progression paths to form pseudo-time series data and performed a series of analyses to characterize dysbiosis and microbiome functional shifts, to infer microbial interactions, and to identify key bacteria associated with CD development. By overcoming the sampling restrictions inherent to slowly progressive diseases, our approach provides a novel way to study microbial community dynamics associated with human chronic diseases.

## Results

### Overview of the developed bioinformatics pipeline

**Fig 1:**
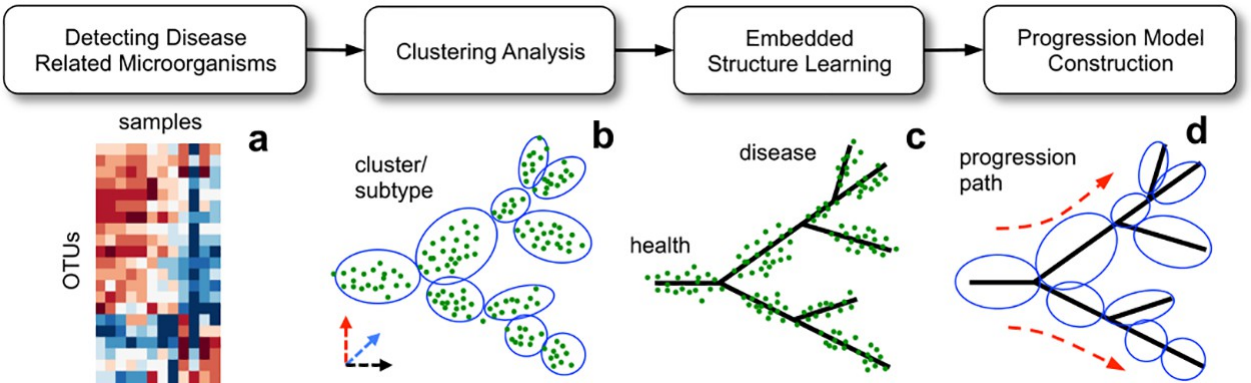
Overview of the proposed bioinformatics pipeline for microbial community dynamics analysis. The pipeline offers an integrated suite of computational tools that allow researchers to identify disease-related microorganisms, stratify samples into clinically relevant subtypes, construct disease progression models, and delineate disease-specific community dynamics at both organism and functional levels.

Fig 1 presents the flowchart of the proposed bioinformatics pipeline for microbial community dynamics analysis. Briefly, given a table of operational taxonomic units (OTUs) that summarizes the microbiome compositions of individuals, either healthy or presenting with different stages of a disease, we first perform feature selection to identify disease-related microorganisms. Then, by using the relative abundances of the selected microorganisms, we perform clustering analysis to group samples with homogenous microbial compositions and conduct embedded structure learning to construct a principal tree to mathematically describe the dynamic changes in microbial compositions associated with disease development. Finally, by using the principal tree as a backbone, we combine the principal-tree and clustering results to build a microbial progression model. See Methods for details. The software and user manual of the proposed bioinformatics pipeline are freely available at www.acsu.buffalo.edu/~yijunsun/lab/MicroDynamics.html.

### Constructing a microbial progression model of Crohn’s disease

To demonstrate the utility of the proposed bioinformatics pipeline, we applied it to a human gut microbiome dataset obtained from a Crohn’s disease study [7]. The dataset contains 312 microbiome samples collected from 49 CD patients and 9 healthy controls (HCs). The disease duration of the CD patients ranged from recent onset to 58 years. By using the Montreal classification system [16], each patient was stratified into one of three disease phenotypes: inflammatory (B1), stricturing (B2), and penetrating (B3). Patients were also classified by bowel lesion location as colonic CD (cCD), ileal CD (iCD), or ileocolonic CD (icCD), and by whether they had undergone resective surgery. See S1 Table for the summary of the study cohort and S2 Table for the detailed clinical information. For each individual, a fecal sample was collected every three months for up to two years, and the V4 hyper-variable region of the 16S rRNA gene was PCR amplified and sequenced, resulting in 90,456,980 reads with an average length of 98 bps. We used the QIIME pipeline [17] for data pre-processing and OTU table construction. Since a sample with an insufficient sequencing depth may not enable accurate estimation of microbial composition, we excluded 37 samples with less than 10^4^ reads from downstream analysis (S1 Fig). By grouping the sequences into OTUs at the 3% distance level, we obtained a total of 77,286 species-level OTUs. See Methods for details.

Since only a small fraction of microorganisms are likely to be involved in disease development, the first step toward progression modeling is to identify disease-related microorganisms. We formulated it as a feature-selection problem for supervised learning and used the disease phenotypes that reflect disease severity as class labels to detect relevant microorganisms. For the purpose of the study, the LOGO algorithm [18] was employed (see Methods). This is one of the most competitive feature-selection algorithms derived to date, with excellent accuracy and computational efficiency. Since the disease progression is defined as the development of B2 or B3 in patients with B1 at diagnosis [12] and there were only 7 patients diagnosed as B3, we combined the samples in the B2 and B3 groups, forming a three-class supervised-learning problem (i.e., HC, B1, and B2/B3). The parameters of LOGO were estimated through ten-fold cross-validation (S2(A) Fig). By applying a cutoff of 0.001 to the obtained feature weights, a total of 172 OTUs were detected to be related to disease development (S2(B) Fig).

**Fig 2:**
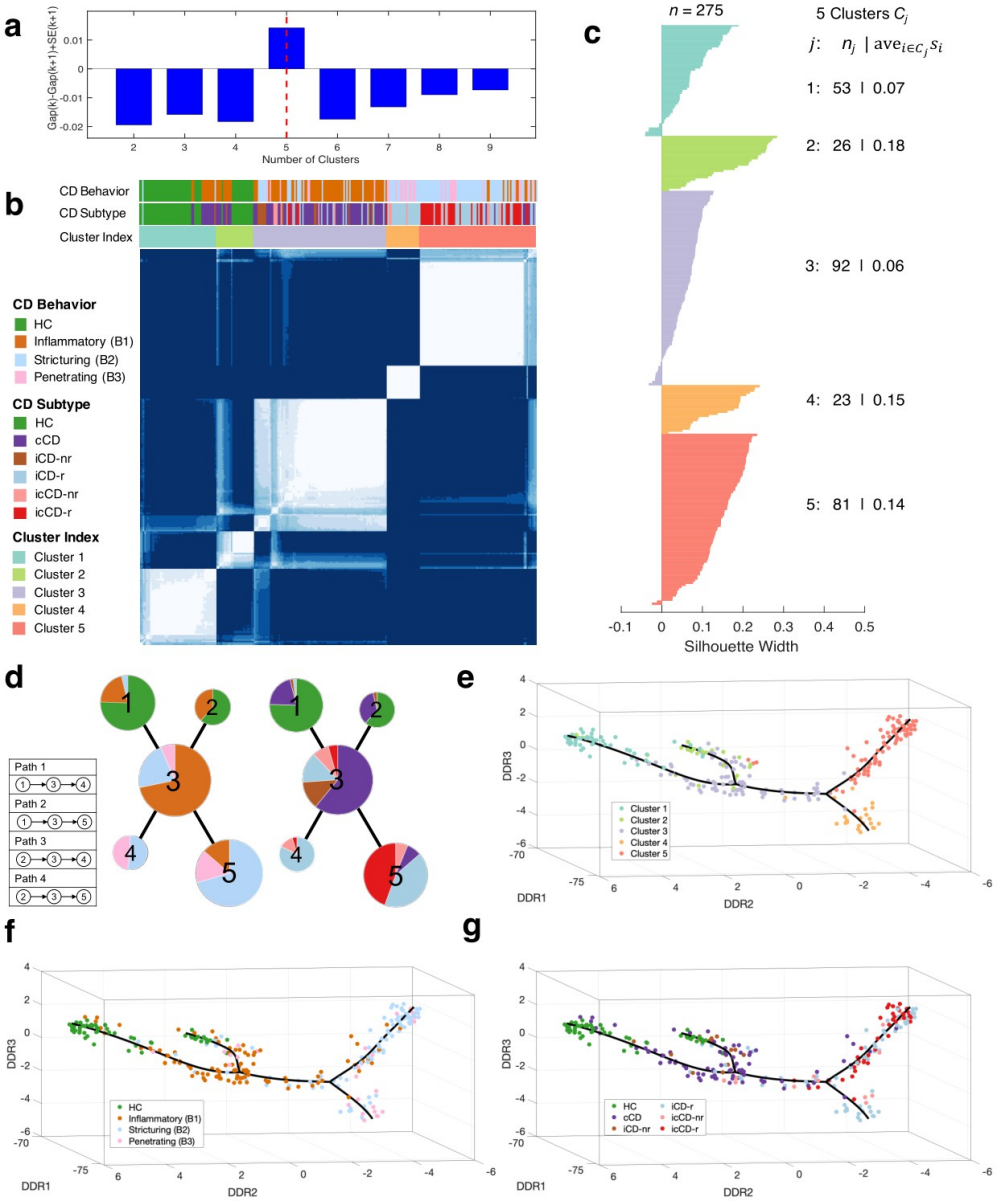
Microbial community dynamic analysis performed on a human gut microbiome dataset (*n* = 275) obtained from a Crohn’s disease study. (**a**) The number of clusters was estimated to be five by gap statistic. (**b**) Resampling-based consensus clustering analysis identified five robust and stable clusters. (**c**) Silhouette width analysis further confirmed the robustness of clustering assignment. A total of 255 of 275 (93%) samples had a positive silhouette width, and the average was equal to 0.1. (**d**) By combining the principal-tree and clustering results, a microbial progression model of Crohn’s disease was constructed, and four progression paths were identified. Each node represents an identified cluster, and the pie chart in each node depicts the percentage of the samples in the node having one of the CD behaviors (left panel) or belonging to one of the CD subtypes (right panel). (**e**-**g**) Visualization analysis provided a general view of sample distribution supported by the selected microorganisms. Each point represents a sample, which was projected onto a three-dimensional space by using the DDRTree method. Each sample was color-coded by its cluster index (**e**), CD behavior (**f**), and CD subtype (**g**), respectively. The solid line represents the constructed principal tree. HC: healthy control, cCD: colonic Crohn’s disease, iCD: ileal Crohn’s disease, icCD: ilealcolonic Crohn’s disease, r/nr: with/without ileocaecal resection.

By using the relative abundance data of the identified OTUs, we next performed a clustering analysis to detect sample groups with homogenous microbial compositions. To this end, the *k*-means method [19] was employed. The number of clusters was estimated to be five by gap statistic [20] (Fig 2A). It is known that using *k*-means may result in a local optimal solution [21]. To obtain a stable and robust clustering assignment, a resampling-based consensus clustering analysis [22] was performed, where *k*-means clustering was repeated 1,000 times and in each time 80% samples were drawn randomly without replacement from the entire dataset. The results of the 1,000 runs were aggregated into a consensus matrix, providing a visual representation of the frequency of two samples being grouped into the same cluster. From the consensus matrix, we can clearly identify five blocks along the anti-diagonal line (Fig 2B), suggesting a reliable data partition. To further assess the clustering robustness, the silhouette width of each sample was calculated, which is defined as the difference between its average similarity with samples in the same cluster and the largest average similarity with samples in different clusters [23]. A cluster with an average silhouette width larger than 0 is generally considered stable. In our analysis, 255 of 275 (93%) samples had a positive silhouette width, the average silhouette widths of the five clusters ranged from 0.06 to 0.18, and the average silhouette width of all the samples equaled to 0.1, demonstrating the stability of the detected clusters (Fig 2C).

After we grouped samples with similar microbial compositions, we next performed an embedded structure learning to construct a principal tree to mathematically describe microbial dynamics and to infer the potential progression relationships of the detected clusters. For the purpose of the study, our recently developed DDRTree algorithm [24] was employed. The basic idea is to fit a given dataset by using a minimum spanning tree with a bounded length (Fig 1C). The method can *automatically* determine the number and presence of branches and is robust against noise, rendering it particularly suitable to detect a complex tree structure hidden in a high-dimensional space. See Methods for details. We used the elbow method [25] to tune the parameters of the DDRTree method (S3 Fig).

Finally, by using the constructed principal tree as a backbone, we combined the clustering and principal-tree results to build a microbial progression model of CD (Fig 2D). We present the constructed model as an undirected graph, where each node represents a cluster, and the node size is proportional to the number of samples in the corresponding cluster. An edge connecting two nodes indicates a possible progressive relationship, and the length of the edge is proportional to the distance of the curve connecting the centers of the two nodes. The pie chart in each node depicts the percentage of the samples in the node having one of the CD behaviors or belonging to one of the CD subtypes. Our modeling analysis revealed a double bifurcating structure with four potential microbiome progression paths, starting from two distinct health-associated clusters and evolving toward two disease endpoints (Fig 2D).

We performed a series of interrogations that provided support for the constructed model. Fig 2E–2G presents the data distribution in a three-dimensional space learned by the DDRTree method. To help with visualization and to put the result into context by referring to previous studies, each sample was color-coded by its cluster index, CD behavior or CD subtype, respectively. We noticed that the overall structure of the constructed model is consistent with the data visualization result (i.e., double bifurcating), suggesting that the model faithfully reflected the data distribution. As mentioned above, changes in CD behavior are part of the natural course of CD, with the disease progression being defined as patients shifting from inflammatory (B1) to a complex behavior (either B2 or B3) [12, 13, 26]. Notably, the identified progression paths accurately reflected the changes in CD behaviors associated with disease progression. As shown in Fig 2D and 2F, the constructed model starts from two health-associated clusters, converges to Cluster 3 that is dominated by samples with an inflammatory behavior, and finally diverges to Clusters 4 and 5 that consist primarily of samples with stricturing and penetrating behaviors. It is worth noting that samples with various CD behaviors (e.g., B1) are present in nearly all the detected clusters (Fig 2D). This is possibly due to the fact that microbial compositions of CD patients are highly unstable and can be heavily influenced by various factors such as dietary changes and medications (discussed below). Changes in bowel lesion location have also been documented to occur during long-term follow-up of CD patients, with initial presentations localized to colon or ileum only and eventually involving both locations [12]. Notably, our microbial progression model captured changes in lesion location (Fig 2D and 2G). Specifically, Cluster 3 was composed mostly of patients with involvement in a single location (cCD and iCD), and Cluster 5 had a large proportion of patients with ileocolonic involvement. This suggests that there are microbial shifts associated with location changes. Our progression model also captured increased disease severity, as measured by the proportion of patients who underwent a resective procedure. Specifically, while there are only 18.8% patients in Cluster 3 having a resection, the proportions are increased to 87.0% and 88.6% for Clusters 4 and 5, respectively (Fig 2D). Taken together, the above results suggest that the constructed model recapitulates the natural history of CD and can provide a representation of the longitudinal progression of dysbiosis during the clinical trajectory of the disease.

### Changes in microbiome alpha diversity along identified progression paths

**Fig 3:**
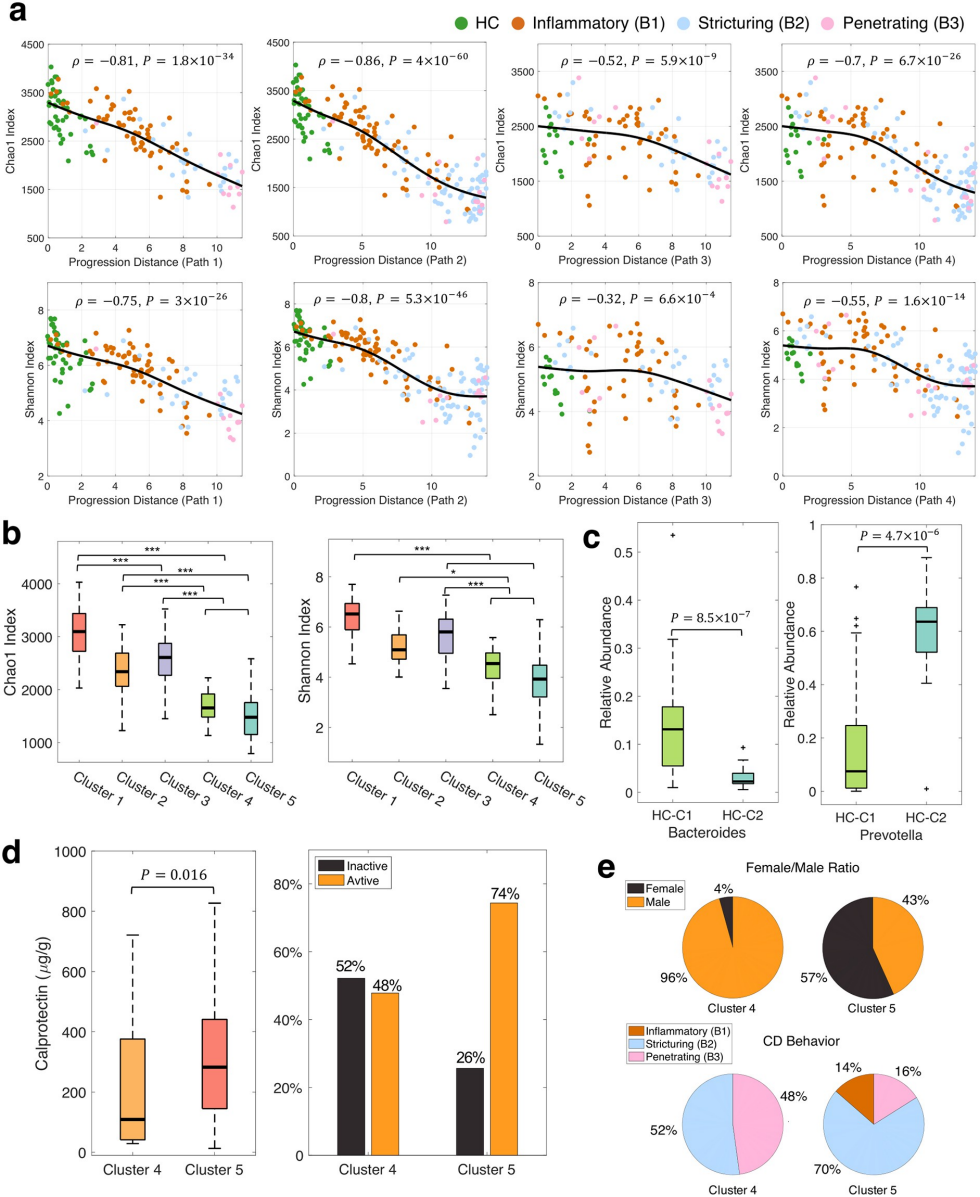
Changes in microbiome alpha diversity along identified progression paths and clinical characteristics of healthy states and disease endpoints. (**a**) Spearman’s rank correlation analysis of alpha diversity as measured by Chao1 index and Shannon index along the four identified progression paths (see Fig 2D). To aid in visualization, each sample was annotated by its clinical behavior. (**b**) Comparison of alpha diversity of five detected clusters. The asterisks indicate the levels of significance determined by ANOVA. *: *p*-value < 0.05, **: *p*-value < 0.01, ***: *p*-value < 0.001. Also see S3 Table. (**c**) Enterotype analysis of the HC samples in Cluster 1 (HC-C1) and Cluster 2 (HC-C2). HC-C1 and HC-C2 correspond to the enterotypes driven by *Bacteroides* and *Prevotella*, respectively. (**d**-**e**) Comparison of clinical characteristics of patients in two disease endpoints (i.e., Clusters 4 and 5). Cluster 5 contained a significantly higher proportion of patients with active inflammation (fecal calprotectin >150 *μ*g/g) compared with Cluster 4 (*p*-value = 0.016, *χ*^2^ test). Clusters 4 and 5 exhibited significantly different female-to-male ratios and CD behavior compositions (*p*-value < 0.01, *χ*^2^ test).

Having depicted the general trend of microbiome shifts during CD development, we assessed the changes in microbiome alpha diversity along the four identified progression paths. We observed that the alpha diversity, as measured by Chao1 [27] and Shannon metrics [28], decreased significantly along each progression path (Fig 3A). We found that Cluster 3, comprised mainly of samples from B1—the early stage of the disease, showed a significantly lower alpha diversity compared with HC Cluster 1 (*p*-value < 0.001, ANOVA test), but not with HC Cluster 2 (Fig 3B). Furthermore, compared with the HC groups and Cluster 3, the clusters of the late-stage CD (Clusters 4 and 5) exhibited a significant reduction in alpha diversity (*p*-value < 0.05, ANOVA test). However, the difference in alpha diversity between Clusters 4 and 5 was not significant. The above results are consistent with the observations from prior studies [29–31] that reported a reduction in microbiome diversity with progressive CD, which provides further support for the validity of the constructed model.

### Characteristics of healthy and disease endpoints

Since the modeled microbiome progression started from two distinct health-associated clusters and evolved toward two disease endpoints, we next evaluated the clinical characteristics of the starting and terminal microbial states. Several studies have demonstrated that human gut microbiota of healthy individuals can be stratified into enterotypes, which are associated with long-term diet and mainly driven by the abundances of *Bacteroides* (termed as ET-B) or *Prevotella* (termed as ET-P) [32, 33]. Notably, in our study, we observed that the HC samples were grouped into two clusters corresponding to ET-B and ET-P, respectively (Fig 3C), and the two clusters converged to the CD-associated Cluster 3 (Fig 2D). This suggests that CD may have two distinct disease origins depending on the enterotypes of individual patients. We noticed that the relative abundance of *Prevotella* in ET-P is much higher than that observed in the original enterotype paper [33]. The discrepancy may be explained by the differences in individual diets, lifestyles, and countries of origin, as reported in [34–36].

The constructed model also depicted two disease endpoints (i.e., Clusters 4 and 5) with distinct clinical characteristics. Specifically, Cluster 5 exhibited a significantly higher proportion of samples showing active inflammation compared with Cluster 4 (*p*-value = 0.016, *χ*^2^ test), as measured by fecal calprotectin > 150*μg*/*g* [37, 38] (Fig 3D). We also observed a gender difference in the two clusters with a female-to-male ratio of 1.31 in Cluster 5 and 0.05 in Cluster 4 (*p*-value ≤ 8.2 × 10^−6^, *χ*^2^ test, Fig 3E). Moreover, there was a significant difference between the two clusters in terms of CD behavior (*p*-value ≤ 2.7 × 10^−3^, *χ*^2^ test, Fig 3E). Specifically, Cluster 5 was dominated by B2 cases—patients with stricturing CD. In contrast, patients with either stricture or penetration were dominant in Cluster 4. To rule out the possibility that the difference in inflammation levels between Clusters 4 and 5 was related to disease behavior, we compared fecal calprotectin levels of patients with the same disease behavior in the two clusters. Our analysis showed that within each CD behavior Cluster 5 always demonstrated a higher portion of samples with active inflammation (S4 Fig) and thus Cluster 5 can be considered as a more severe phenotype. In summary, our analysis suggests that there may be two forms of late-stage CD, with different microbiome compositions, inflammation severities, CD behaviors and subtypes (Fig 2D).

### Characterizing overall dysbiotic shifts during CD progression

**Fig 4:**
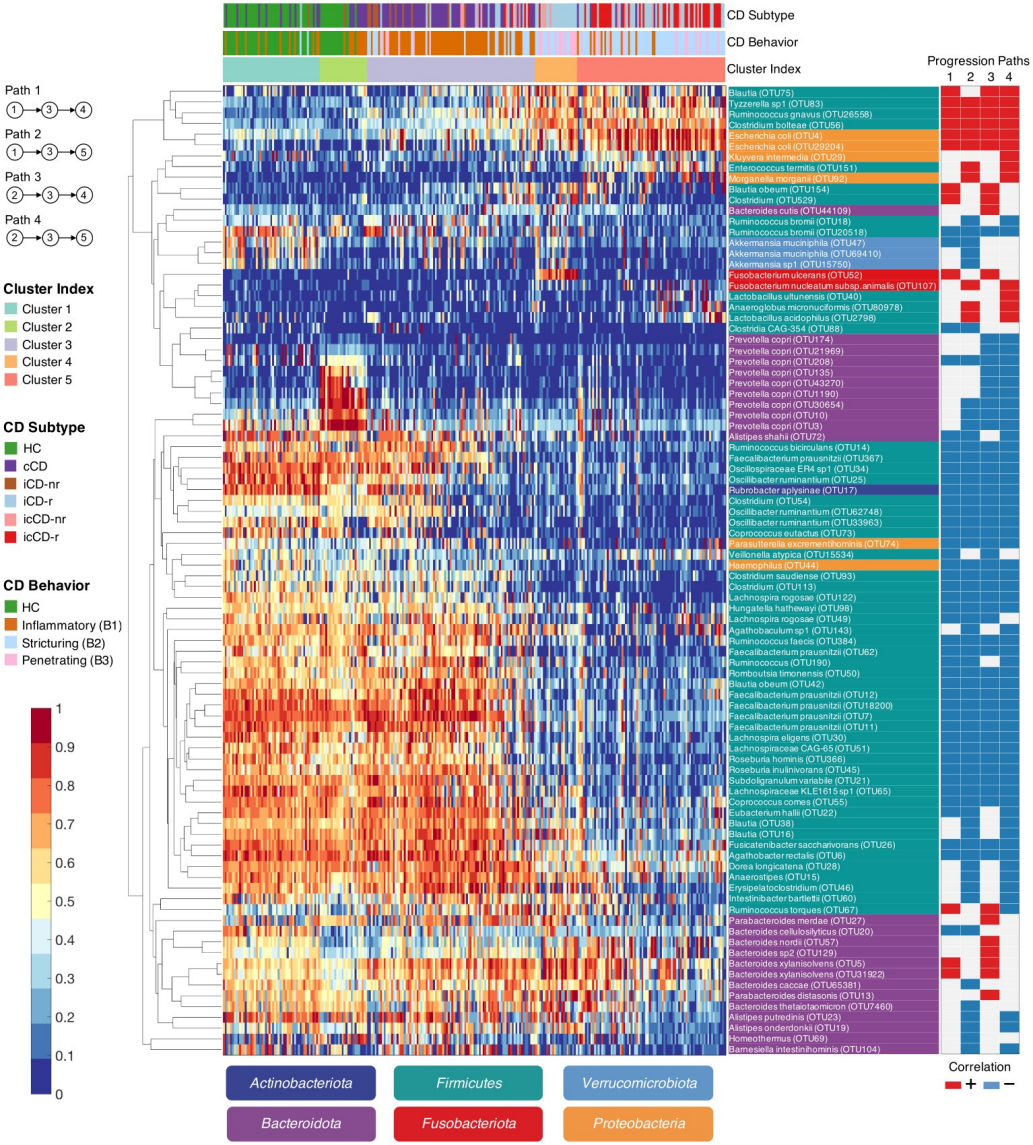
Heatmap of microorganisms for which the relative abundances were detected to be highly correlated with at least one of the four identified CD progression paths. Each row represents an OTU, and each column represents a sample. The samples were first ordered by cluster labels and then by progression distances. For the purpose of visualization, the relative abundance of each OTU was log-transformed and scaled into the range of [0, 1]. See S5 Fig for additional details.

In the progression modeling analysis, we performed supervised learning to detect disease-related microorganisms. However, due to the use of the ℓ_1_ regularization (see Eq (6)), if multiple microorganisms had similar microbial profiles across samples, only one microorganism was retained (that is, we intended to construct a parsimonious model to minimize the chance of overfitting). To comprehensively search for disease-related microorganisms, we performed a Spearman’s rank test to detect OTUs that showed significant changes in relative abundance along at least one identified progression path. We used the DS-FDR method [39] to control the false discovery rate and filtered out OTUs with average relative abundance < 0.001 and Spearman’s rank correlation coefficient |*ρ*| < 0.3 (i.e., those with a weak or no correlation). At an FDR of 0.01, a total of 90 species-level OTUs were retained (Fig 4 and S5 Fig).

Consistent with previous findings [7, 40, 41], we observed an overall decrease of beneficial bacteria including *Faecalibacterium prausnitzii*, *Roseburia*, *Subdoligranulum* and *Lachnospiraceae*, as well as members of *Ruminococcus* and *Oscillospiraceae* as disease severity progressed (S6 Fig). By producing butyric acid, the beneficial bacteria, such as *F. prausnitzii*, may protect the host by up-regulating anti-inflammatory cytokines [42]. Thus, the reduction of these clades may impair the ability of the host to repair the epithelium and regulate inflammation. In contrast, the relative abundances of pro-inflammatory bacteria, including *Escherichia coli* and *Ruminococcus gnavus*, were significantly increased along the disease progression paths (S7 Fig). Our data confirms previous findings that suggested that *E. coli* and *R. gnavus* may play a role in CD development [43]. In addition, OTUs classified as *Tyzzerella sp*. and *Clostridium bolteae* were found by our pipeline to be associated with CD progression through all paths (S7 Fig).

Importantly, our analysis also detected path-specific microbial variations. A decrease in *Prevotella copri* was associated with the path starting from ET-P, while *Clostridia CAG-354*, *Bacteroides cellulosilyticus* and *Akkermansia muciniphila* decreased with disease progression from ET-B. Notably, *Ruminococcus torques*, which is known to degrade gastrointestinal mucin [44, 45] and is more frequently found in relatives of CD patients compared with healthy individuals [46], was positively correlated with the disease progression paths leading to Cluster 4. *Fusobacterium ulcerans*, which has been previously isolated from skin ulcers, was also increased in a specific manner along progression paths 1 and 3 leading to Cluster 4. Microbial changes leading to Cluster 5—a severe disease status—included a decrease in the beneficial gut commensal *Anaerostipes*, which may protect against colon cancer by producing butyric acid [47], and an increase in the oral commensals *Fusobacterium nucleatum subsp. animalis*, *Lactobacillus acidophilus* and *Anaeroglobus micronuciformes* along both paths 2 and 4, leading to Cluster 5.

### Inferring microbial interaction networks associated with CD progression

**Fig 5:**
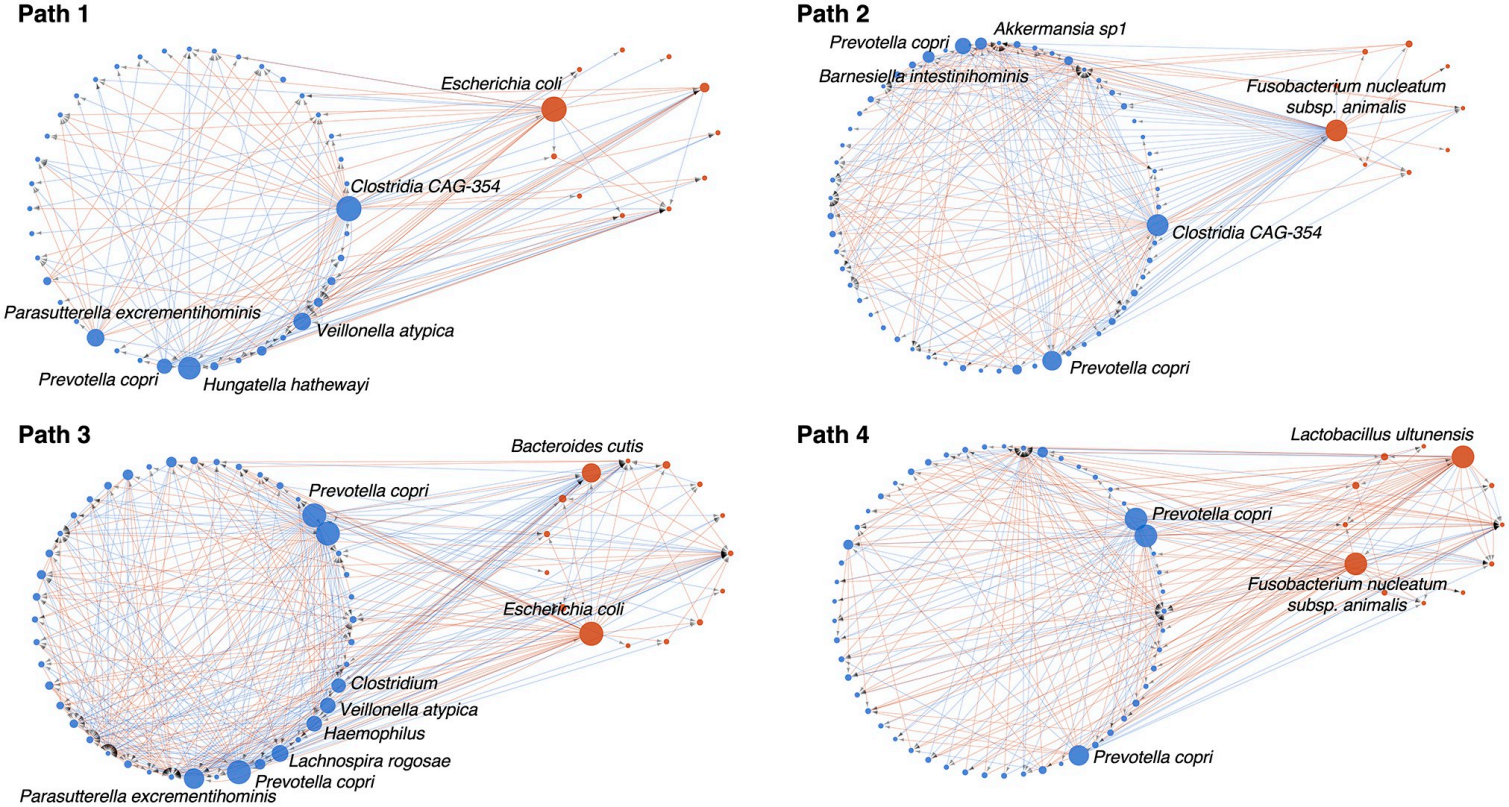
Microbial interaction networks inferred by the gLV method applied to pseudo-time series data recovered from four identified CD progression paths. Each node represents an OTU, its size is proportional to the number of edges directed out of the node (i.e., out-degree), and its face color represents the sign of the correlation of the relative abundance of the OTU with a progression path (red: positive, blue: negative). Only the nodes with out-degrees larger than 10 were annotated. Since compositionality was not considered in the analysis, artificial links might arise. See S9 Fig for detailed annotations.

Once a microbial progression model was constructed, we projected each sample back onto the identified progression paths. Here, the projection of a sample was defined as a point on a progression path that is closest to the sample. By using the healthy controls as the baseline, the static samples were ordered along a path according to the extent to which the disease progressed from an inflammatory phenotype toward intestinal stricture and penetration (S8 Fig). The ordered samples can be viewed as *pseudo-time series* data, which provides a unique opportunity to perform a microbial interaction network analysis to identify key bacteria potentially responsible for the alterations of microbiota associated with disease development. In this study, we used the generalized Lotka-Volterra (gLV) method [48, 49] to infer pairwise interactions between microorganisms (see Methods). Following the work of [50], we evaluated the influence of each microorganism (or node) affecting others on the network by its out-degree—the number of edges directed out of the node. We found that a decrease in *Prevotella copri* was an important event associated with disease development along all paths (Fig 5 and S9 Fig). Our analysis also revealed that a decrease in *Parasutterella excrementihominis* and *Veillonella atypica* and an increase in *E. coli* are key events in the progression toward Cluster 4, while the progression leading to Cluster 5 appears to be primarily driven by an increase in *F. nucleatum subsp. animalis*.

### Characterizing functional shifts associated with CD progression

The constructed progression model also enabled us to investigate how the shifts in functional potential of the microbiome were associated with disease development. To this end, we applied PICRUSt2 [51] to predict the functional content of microbial communities and performed a Spearman’s rank correlation test to identify KEGG pathways that showed significant changes in pathway activities along at least one progression path (see Methods). As with the dysbiosis analysis, we employed the DS-FDR method [39] to control the false discovery rate and filtered out the functional pathways with average relative abundance < 0.001 and Spearman’s rank correlation coefficient |*ρ*| < 0.3. At an FDR of 0.01, a total of 101 KEGG pathways were identified (S10 and S11 Figs and S4 Table). Consistent with previous studies [52–55], we found that the activities of pathways such as galactose metabolism, pentose and glucuronate interconversions, sulfur metabolism, glyoxylate and dicarboxylate metabolism, nitrogen metabolism, and phenylalanine metabolism were significantly increased with CD severity. Conversely, pathways including fatty acid biosynthesis, phenylalanine, tyrosine and tryptophan biosynthesis, and D-glutamine and D-glutamate metabolism were negatively correlated with the disease progression paths. Through comparative analysis of pathway activities along different progression trajectories, we found that progression paths leading to Cluster 4 were associated with decreased amino acid and nucleotide metabolism along with increased metabolism of several carbohydrates, glycan degradation and primary and secondary bile acid biosynthesis, while the progression paths that end at Cluster 5 were linked to a decline of antimicrobial biosynthesis and an enrichment in glutathione metabolism, and xylene and dioxin degradation. Path 4 in particular was associated with an increase in two-component systems, ABC transporters, the phosphotransferase system, and the butyrate and propionate metabolic pathways. These results highlight the ability of the proposed bioinformatics pipeline to identify microbiome functional shifts along disease progression paths toward distinct disease phenotypes.

### Longitudinal microbiome shifts in individual subjects during disease progression

**Fig 6:**
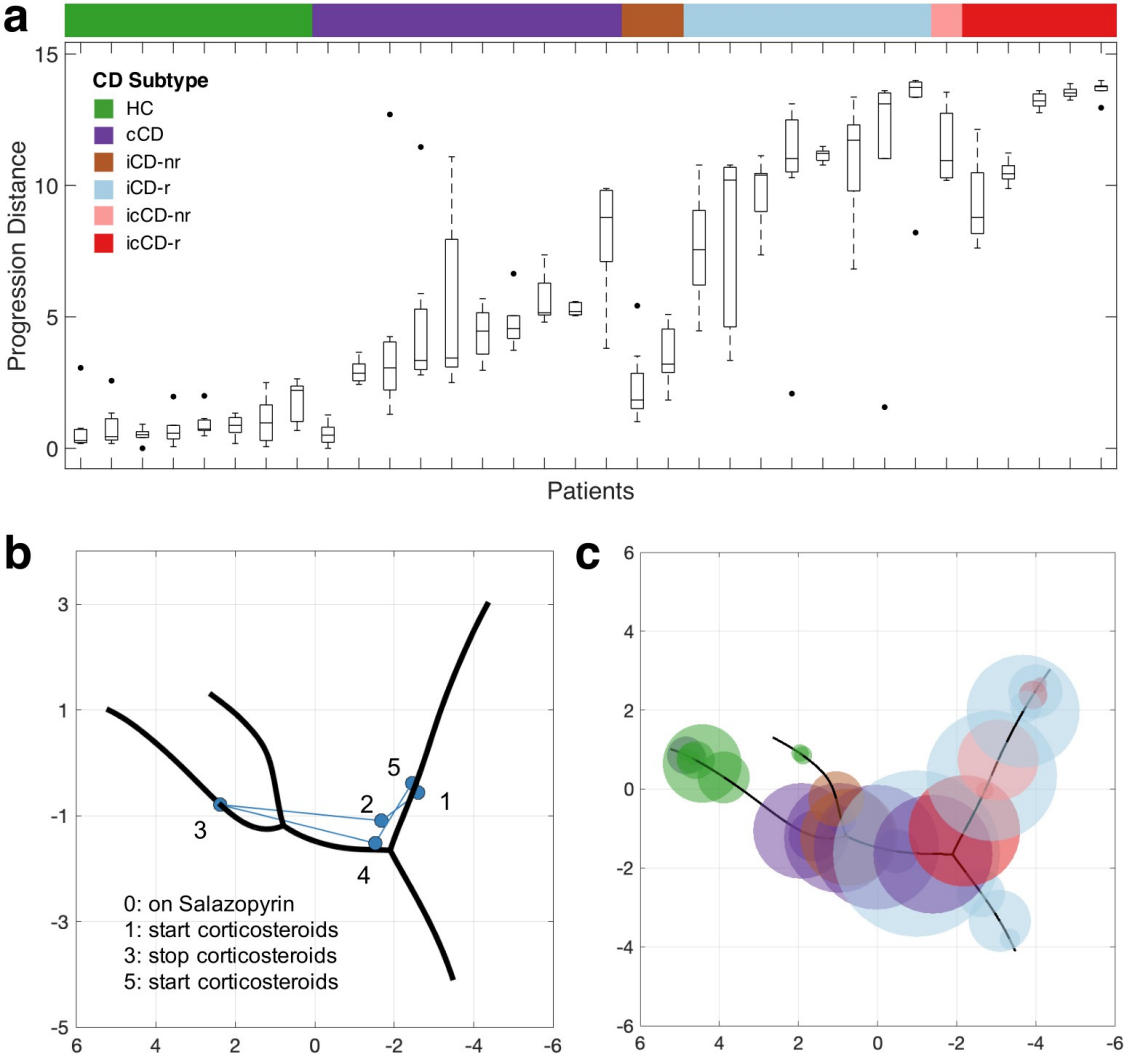
Microbial community dynamics analysis of individual patients. (**a**) The progression distances of the samples collected from individual participants over a two-year period. The participants were first ordered by CD subtypes and then by median progression distances. (**b**) The microbiome composition of a patient was significantly altered by medication. Sample 0 contained only a few hundreds of reads and thus was omitted. (**c**) Samples collected from a two-year study provided only a partial picture of microbial community dynamics associated with disease development. Each circle represents a patient, the face color represents the CD subtype, and the radius equals 1.5 MAD of the progression distances of the samples collected from the patient. MAD: median absolute deviation.

The study cohort was collected from participants every three months for up to two years, which provided us with an opportunity to examine variations in the microbiome of individual patients across the sampling period. Since it is not reliable to estimate variations using a small number of samples, we excluded from the analysis the individuals with < 5 serial samples. In total, data from 34 participants were examined. Since both ET-B and ET-P can be used as the disease origin, the progression distance of a sample could vary depending on the origin used. To address this issue, we scaled the curve distance between Clusters 2 and 3 (i.e., the shorter branch) by a constant of 1.66 in downstream analysis so that the calculation of the progression distance of a given sample is independent of the disease origin used. Our analysis showed that CD samples significantly deviated from HCs (*p*-value < 0.001, Student’s t-test, Fig 6A). Specifically, the icCD-r cases attained the largest progression distances, in line with reported clinical severity, followed by icCD-nr and iCD-r. We can also see that the microbial communities of healthy individuals are much more stable than those of CD patients. In addition to physiological conditions, other factors (e.g., dietary changes and medications) can also significantly alter the human gut microbiome. For example, in a patient who was diagnosed with cCD and placed on corticosteroids, we found that in the period of receiving the medication the gut microbiome moved backward along the progression path toward the healthy microbiome, but after corticosteroids were stopped, the microbiome moved forward along the progression path toward the initial microbial status, at which point corticosteroids were again administered (Fig 6B). This result indicates that the constructed progression model correlates well with the clinical trajectory of CD and supports the potential of such tools to monitor treatment responses and disease remission.

To further assess the sample variation of each patient, we computed the median absolute deviation (MAD) of the progression distances of the samples from each patient (1.5 MAD is a robust measure of one standard deviation) and mapped the samples back onto the progression model (Fig 6C). We found that the radii of the circles representing CD patients ranged from 0.13 to 2.13 (average: 0.83), which equals to 5.9%–9.1% of the total length of the progression path, respectively. This analysis shows that while the gut microbiome of CD patients is highly unstable and influenced by various factors, the samples collected from a two-year longitudinal study provided *only* a partial picture of the progressive clinical course of CD. This underscores the importance of the development of novel approaches, such as the bioinformatics pipeline proposed in this study, to overcome the sampling limitations that impede longitudinal studies of microbiome-related chronic diseases.

## Discussion

As with any biological system, a microbial community is a dynamic system undergoing constant change in response to internal and external stimuli. The composition of the human gut microbiota, for example, can be modulated by the introduction or extinction of particular microbial groups, or by a change in population structure caused by various factors [56]. In turn, such changes can have significant implications for human health [57]. The delineation of system dynamics of a microbial community can provide a wealth of insights not accessible through a static experiment, and lay a critical foundation for the development of probiotic, prebiotic, antibiotic, and other strategies to manipulate the microbiome. However, due to the difficulty in obtaining longitudinal samples, most existing microbiome studies have been cross-sectional and largely descriptive. Here, we present a novel computational strategy that leverages massive static sample data to study microbial dynamics associated with chronic human disease development. We applied the developed pipeline to a Crohn’s disease microbiome dataset and constructed one of the first microbial progression models of the disease. Our analysis revealed that CD may have two disease origins depending on the enterotypes of individual patients, and two disease endpoints with distinct clinical characteristics and microbial compositions. Since there is currently no established progression model for comparison, model validation poses a challenge. Our strategy was to align the model with established clinical and molecular traits. Our analysis suggested that the constructed model recapitulated the longitudinal progression of microbial dysbiosis during the known clinical trajectory of CD.

This study has several limitations worth discussing. It has been reported that the compositional nature of microbiome data could induce biases in data analysis [58–60]. While there are several strategies (e.g., modeling with data after centered log-ratio (CLR) transformation) that can be used to alleviate the issue [59], the analysis of compositional microbiome data remains a challenge [60]. Due to the lack of microbial biomass data of samples, our approach assumed that there was no significant variation in absolute abundances between samples. Thus, the compositionality was not factored into the modeling. Out of interest, we performed an experiment using CLR transformed data for modeling, and we observed a similar double bifurcating structure. However, we should point out that in the microbial interaction network analysis, artificial links might arise since compositionality was not considered. In the software package, we provided users with an option to use CLR transformed data for the proposed analysis. As more data becomes available, we will perform in-depth analysis to assess potential biases that compositional data might introduce into a model. In this study, we used the *k*-means method to detect patient groups with homogenous microbial compositions. While *k*-means is one of the most widely methods for clustering analysis, there are several other methods that might be more suitable for microbiome data sets (e.g., Dirichlet multinomial mixtures [61] and partition around medoids [62]). Another limitation of the study is that the model was derived from a dataset with a relatively small sample size. When larger datasets become available, the development of robust models encompassing the microbiome variability across individuals and populations will become possible. In this study, we used the sequence data obtained from the V4 hyper-variable region of the 16S rRNA gene for OTU table construction, which may not provide sufficient taxonomic resolution at the species level and can affect model resolution. A possible way to address the issue is to use whole metagenome or full-length 16S rRNA gene sequence data to estimate microbial compositions, which could significantly refine constructed models. We should emphasize that the constructed model ultimately needs to be verified through wet-lab experiments. However, interrogation of a computational model will allow researchers to generate and test novel hypotheses *in silico* and help to prioritize resources and inform focused and detailed investigations experimentally.

We expect that our approach will find wide applications. Although here we focused on Crohn’s disease, the approach can be used to study other microbe-related chronic diseases, where the lack of longitudinal data is a *ubiquitous* problem. Compared to resource-intensive or impractical time-course studies, it is much easier to conduct a cross-sectional study; researchers only need to be concerned with recruiting patients presenting various stages of a disease, and the recent development of sequencing technology has already made large-scale sequencing projects feasible. The application of our approach to large cross-sectional populations will significantly advance our understanding of microbiome dynamics during chronic disease development and help to identify novel diagnostic and therapeutic strategies.

## Materials and methods

### Data pre-processing and OTU table construction

We used the QIIME pipeline (v1.9.0) [17] for data pre-processing and OTU table construction. Specifically, we first removed low-quality reads by filtering out sequences that contained ambiguous bases, had a Phred quality score less than 3, or had more than three consecutive low-quality base calls. A total of 90,456,980 sequences were retained for further analysis. Then, we performed a taxonomy-independent analysis using USEARCH [63] to group sequences into OTUs at the 3% distance level, and removed chimeric sequences using UCHIME [64]. We calculated the relative abundance of each sample by dividing the number of reads in each OTU by the total read counts in the sample. We added a small constant 10^−6^ to the relative abundances and performed a 10-base logarithmic transformation [65]. We performed the taxonomy annotation of each OTU by using BLAST [66] against the Genome Taxonomy Database (v86) [67], and conducted a functional analysis by using PICRUSt2 [51]. The functional analysis yielded 8,602 KEGG orthologies, which were grouped into 204 KEGG pathways [68] by MinPath [69].

### Bioinformatics pipeline for microbial community dynamics analysis

#### Feature selection to identify disease related microorganisms

We used the LOGO algorithm [18] to identify disease-related microorganisms. It represents one of the most competitive feature-selection algorithms derived to date, with excellent accuracy and computational efficiency. The basic idea is to decompose a complex nonlinear problem into a set of locally linear ones through local learning, and then learn feature relevance globally within the large margin framework. Below, we present a detailed description of the method.

Let ${\left\{\left({\text{x}}_{n},y_{n}\right)\right\}}_{n=1}^{N}$ be a dataset, where **x**_*n*_ is the *n*-th data sample and *y*_*n*_ is the corresponding label. We aim to select a subset of features so that the class labels of unseen samples can be correctly predicted. We start by defining the margin of **x**_*n*_. Given a distance function, we find two nearest neighbors of **x**_*n*_, one from the same class (called nearest hit or NH), and the other from a different class (called nearest miss or NM). The margin of **x**_*n*_ is defined as *ρ*_*n*_ = *d*(**x**_*n*_, NM(**x**_*n*_)) − *d*(**x**_*n*_, NH(**x**_*n*_)), where *d*(⋅) is a distance function. In this study, we used the Manhattan distance to define the margin, while other distance function can also be used. By the large margin theory [70], a classifier that minimizes a margin-based error function usually generalizes well on unseen test data. Let **w** ≥ 0 be a feature weight vector, where the magnitude of each element represents the relevance of the corresponding feature. Our goal is to find a weighted subspace specified by **w** so that a margin-based error function in the induced space is minimized.

The margin of **x**_*n*_, computed with respect to **w**, is given by

$$\begin{array}{c} \rho_{n}\left(\text{w}\right)=d\left({\text{x}}_{n},\text{NM}\left({\text{x}}_{n}\right)|\text{w}\right)-d\left({\text{x}}_{n},\text{NH}\left({\text{x}}_{n}\right)|\text{w}\right)≜{\text{w}}^{T}{\text{z}}_{n}\;, \end{array}$$ (1)

where **z**_*n*_ = |**x**_*n*_ − NM(**x**_*n*_)| − |**x**_*n*_ − NH(**x**_*n*_)|, and |⋅| is an element-wise absolute operator. A major issue with the above margin definition is that the nearest neighbors of a given sample are unknown before learning. To account for the uncertainty in defining local information, we develop a probabilistic model, where the nearest neighbors of a given sample are treated as *hidden* variables. Following the principles of the expectation-maximization algorithm [71], we estimate the margin by computing the expectation of *ρ*_*n*_(**w**) via averaging out the hidden variables:

$$\begin{array}{c} \begin{array}{c} \rho_{n}\left(\text{w}\right)={\text{w}}^{T}(\sum_{i\in M_{n}}P\left({\text{x}}_{i}=\text{NM}\left({\text{x}}_{n}\right)|\text{w}\right)|{\text{x}}_{n}-{\text{x}}_{i}|-\sum_{i\in H_{n}}P\left({\text{x}}_{i}=\text{NH}\left({\text{x}}_{n}\right)|\text{w}\right)\left|{\text{x}}_{n}-{\text{x}}_{i}\right|)≜{\text{w}}^{T}{\bar{\text{z}}}_{n}\;, \end{array} \end{array}$$ (2)

where $M_{n}=\left\{i:1\leq i\leq N,y_{i}\neq y_{n}\right\}$, $H_{n}=\left\{i:1\leq i\leq N,y_{i}=y_{n}\right\}$, and *P*(**x**_*i*_ = NM(**x**_*n*_)|**w**) and *P*(**x**_*i*_ = NH(**x**_*n*_)|**w**) are the probabilities of sample **x**_*i*_ being the nearest miss or hit of **x**_*n*_, respectively. The probabilities are estimated via the standard kernel density estimation:

$$\begin{array}{c} P({\text{x}}_{i}=\text{NM}\left({\text{x}}_{n}\right)|\text{w})=\frac{K(d({\text{x}}_{n},{\text{x}}_{i}|\text{w}))}{\sum_{j\in M_{n}}K\left(d\left({\text{x}}_{n},{\text{x}}_{j}|\text{w}\right)\right)},\forall i\in M_{n}\;, \end{array}$$ (3)

and

$$\begin{array}{c} P\left({\text{x}}_{i}=\text{NH}\left({\text{x}}_{n}\right)|\text{w}\right)=\frac{K(d({\text{x}}_{n},{\text{x}}_{i}|\text{w}))}{\sum_{j\in H_{n}}K(d({\text{x}}_{n},{\text{x}}_{j}|\text{w}))},\forall i\in H_{n}\;, \end{array}$$ (4)

where *K*(⋅) is a kernel function. In this study, we employed the Epanechnikov kernel [72], given by

$$\begin{array}{c} K(d({\text{x}}_{n},{\text{x}}_{i}|\text{w}))=\{\begin{array}{cc} \frac{3}{4}(1-(\frac{d({\text{x}}_{n},{\text{x}}_{i}|\text{w})}{d({\text{x}}_{n},{\hat{\text{x}}}_{k+1}|\text{w})}{)}^{2}) & \;\text{if}\;\frac{d({\text{x}}_{n},{\text{x}}_{i}|\text{w})}{d({\text{x}}_{n},{\hat{\text{x}}}_{k+1}|\text{w})}\leq 1\\ 0 & \text{otherwise}, \end{array} \end{array}$$ (5)

where ${\hat{\text{x}}}_{k+1}$ is the (*k* + 1)-th nearest neighbor of **x**_*n*_ in a feasible set. To reduce the number of parameters to be tuned, we simply set *k* = 10.

Once we define the margins, we solve the problem of learning feature weights within the large-margin framework. Specifically, we perform the estimation using the logistic-regression formulation, and obtain the following optimization problem:

$$\begin{array}{c} \underset{\text{w}}{\text{min}}\;\sum_{n=1}^{N}\;\text{log}\;(1+\text{exp}(-{\text{w}}^{T}{\bar{\text{z}}}_{n}))+\lambda \|\text{w}{\|}_{1},\;\text{subject}\;\text{to}\;\text{w}\geq 0\;. \end{array}$$ (6)

Here, we impose an *ℓ*_1_ constraint on **w** to achieve a sparse solution [73], and λ is a regularization parameter that can be estimated by using ten-fold cross-validation. Problem (6) can be solved iteratively. Briefly, we first make a guess on **w**. Then, we find the nearest neighbors of each sample and compute ${\bar{\text{z}}}_{n}$. Finally, we update **w** by solving Problem (6). The iterations are carried out until convergence.

#### Embedded structure learning to delineate microbial community dynamics

After we identified groups of samples with similar microbiome compositions, we built a model to mathematically describe the microbial dynamics associated with disease development. To this end, principal curve fitting methods were used. Formally, a principal curve is a nonlinear generalization of the first principal-component line passing through data cloud. In the last decade, a dozen methods have been developed for principal curve fitting. However, they are generally limited to learn a curve that is embedded in a low-dimensional space and does not intersect itself, which is quite restrictive for real applications. We have recently developed a new graphic model-based method, referred to as DDRTree [24], that addresses some limitations of prior work.

Let **X** = [**x**_1_, ⋯, **x**_*N*_] be a dataset in the input space $X\subset R^{D}$ and **x**_*n*_ be the *n*-th sample. We assume that the structure to be learned lies in a latent space $Y\subset R^{d}$ with *d* ≪ *D*, and use an undirected graph *G* = (*V*, *E*) to represent the structure, where *V* = {*v*_1_, ⋯, *v*_*N*_} is a set of vertices and *E* is a set of edges. We introduce a set of latent variables **Z** = [**z**_1_, ⋯, **z**_*N*_] to explicitly represent the graph, and associate **z**_*n*_ with vertex *v*_*n*_. Following the work of Gaussian mixture models [21], we assume that the observed data **X** are generated through a random process. Specifically, we first randomly select a data point residing on the graph, then corrupt the data with some random noise, and finally map the corrupted data back onto the input space. Let **Y** = [**y**_1_, ⋯, **y**_*N*_] be the corrupted data. Our goal is to find the latent variables **Z** and a mapping function $f:R^{d}\to R^{D}$ that projects data in the latent space back onto the input space so that the reconstruction error is minimized. Without explicitly specifying a form for *f*, it is generally difficult to learn the structure of a graph. For the purpose of the study, we use a linear mapping function *f* (**y**_*n*_) = **Wy**_*n*_, where $\text{W}\in R^{D\times d}$ is a projection matrix and **W**^*T*^**W** = **I**. To avoid the difficulty of learning a general graph, we consider *G* to be a minimum spanning tree (MST) [74], where the costs of the edges are defined to be the squared Euclidean distances of the latent variables. By combining all the above considerations, we obtain the following formulation:

$$\begin{array}{c} \begin{array}{cc} \underset{\text{W},\text{Z},\text{Y},\left\{b_{ij}\right\},\left\{p_{ij}\right\}}{\text{min}} & \sum_{n=1}^{N}\|{\text{x}}_{n}-\text{W}y_{n}{\|}^{2}+\gamma \sum_{i,j=1}^{N}p_{ij}(\|y_{i}-{\text{z}}_{j}{\|}^{2}+\sigma \;\text{log}\;p_{ij})\\ \text{subject}\;\text{to} & \;\sum_{i,j=1}^{N}b_{ij}{\left\|{\text{z}}_{i}-{\text{z}}_{j}\right\|}^{2}\leq \ell ,{\text{W}}^{T}\text{W}=I,\sum_{j=1}^{N}p_{ij}=1,p_{ij}\geq 0,\;\forall i,j, \end{array} \end{array}$$ (7)

where *p*_*ij*_ is the probability of assigning **y**_*i*_ to **z**_*i*_, *σ* is a parameter for soft assignment using negative entropy regularization [75], *γ* is a parameter that controls the tradeoff between the data reconstruction error and the quantization error, and *b*_*ij*_ is constrained to be a feasible solution of an MST that takes a value of 1 if (*v*_*i*_, *v*_*j*_) ∈ *E* and 0 otherwise. The above formulation can be interpreted as fitting a dataset by using an MST with a length bounded by *ℓ* (see Fig 1C). For ease of optimization, we moved the length constraint to the objective function:

$$\begin{array}{c} \begin{array}{cc} \underset{\text{W},\text{Z},\text{Y},\left\{b_{ij}\right\},\left\{p_{ij}\right\}}{\text{min}} & \sum_{n=1}^{N}\|{\text{x}}_{n}-\text{W}y_{n}{\|}^{2}+\frac{\lambda }{2}\sum_{i,j=1}^{N}b_{ij}{\left\|{\text{z}}_{i}-{\text{z}}_{j}\right\|}^{2}+\gamma \sum_{i,j=1}^{N}p_{ij}(\|y_{i}-{\text{z}}_{j}{\|}^{2}+\sigma \;\text{log}\;p_{ij})\\ \text{subject}\;\text{to} & \;{\text{W}}^{T}\text{W}=I,\sum_{j=1}^{N}p_{ij}=1,p_{ij}\geq 0,\;\forall i,j, \end{array} \end{array}$$ (8)

where λ is a regularization parameter. For the purpose of data visualization, we projected the samples onto a three-dimensional space (i.e., *d* = 3). In order not to tune too many parameters, following the work of [24], we set *γ* = 2 and estimated the kernel width *σ* and the regularization parameter λ by using the elbow method [25]. Problem (8) can be efficiently solved by using alternating structure optimization [24, 76]. Briefly, we first fix {*b*_*ij*_} and {*p*_*ij*_} and find a solution for **W**, **Z**, and **Y** via convex optimization. Then, we fix **W**, **Z**, and **Y** and find a solution for {*b*_*ij*_} by solving an MST problem using Kruskal’s method [77] and solve {*p*_*ij*_} analytically. The two steps iterate until convergence.

#### Constructing a microbial progression model

We combined the clustering and principal-tree results to build a progression model and extract progression paths. We represented a progression model as an undirected graph, where the vertices were the centroids of the clusters identified in the cluster analysis and they were connected based on the progression trend inferred from the principal curve. Specifically, we first projected each sample back onto the principal tree, and then extracted the progression paths by finding the shortest path from a designated root vertex to all the leaf vertices of the principal tree. In this study, we used the leaf node of the healthy control samples as the root vertex to represent the origin of the disease. By using the same procedure, we mapped the centroids of the clusters onto the principal tree and constructed an undirected graph. Two projected centroids were connected if there were no other centroids between them along a progression path, and the length of the edge was proportional to the curve distance of the two centroids measured along the progression path.

### Microbial interaction network analysis

By using the pseudo-time series data recovered from the identified progression paths, we built generalized Lotka-Volterra (gLV) models to study microbial interactions associated with disease development. The gLV model has been successfully applied to several longitudinal studies to uncover pairwise interactions between microorganisms and to identify key bacteria possibly responsible for the alterations of microbiota associated with the development of a disease [48, 49]. Let *x*_*i*_(*t*) be the relative abundance of the *i*-th OTU, measured at time *t*, 1 ≤ *t* ≤ *T*. A gLV model can be represented as a set of first-order ordinary differential equations, given by

$$\begin{array}{c} \frac{dx_{i}\left(t\right)}{dt}=x_{i}\left(t\right)(\alpha_{i}+\sum_{j=1}^{J}\beta_{ij}x_{j}\left(t\right)),1\leq i\leq J, \end{array}$$ (9)

where *J* is the number of OTUs, *α*_*i*_ is the growth rate of the *i*-th OTU, and *β*_*ij*_ is the strength of the pairwise interaction between the *i*-th and *j*-th OTUs. To simulate a biologically realistic ecological system where interacting species may have a wide range of relationships including competition, cooperation, or neutralism, we assume that the growth rates are positive (i.e., *α*_*i*_ > 0) and the self-intersection rates are negative (i.e., *β*_*ii*_ < 0) [78]. Dividing both sides of Eq (9) by *x*_*i*_(*t*) yields

$$\begin{array}{c} \frac{d\;\text{ln}\;x_{i}\left(t\right)}{dt}=\alpha_{i}+\sum_{j=1}^{J}\beta_{ij}x_{j}\left(t\right),1\leq i\leq J\;, \end{array}$$ (10)

which can be further approximated as a linear system, given by

$$\begin{array}{c} \frac{d\;\text{ln}\;x_{i}\left(t\right)}{dt}\approx {\left(\text{ln}\;x_{i}\left(t\right)\right)}^{\prime }\approx \alpha_{i}+\sum_{j=1}^{J}\beta_{ij}x_{j}\left(t\right),1\leq i\leq J\;, \end{array}$$ (11)

where (ln *x*_*i*_(*t*))′ is the gradient of ln *x*_*i*_(*t*) at time *t*. We used a two-step estimation procedure [79] to solve the above linear system. Specifically, we first estimated the log-transformed relative abundances and the corresponding gradients of each OTU along a progression path using cubic smoothing spline, and then estimated the variables of a gLV model using Bayesian Adaptive Lasso [79] implemented by the MDSINE package [78] with default settings. Once the linear system was solved, we built a gLV interaction network of the OTUs for each identified disease progression path.

### Alpha diversity estimation

We used alpha diversity, specifically Chao1 index [27] and Shannon index [28], to assess the species richness of the gut microbial communities of individual patients. Since the estimation of alpha diversity can be biased for communities with different sequencing depth [80], we performed a rarefaction analysis by sampling 10,000 reads from each community and then calculated the corresponding alpha diversity. The process was repeated 1,000 times and the average value was reported.

### Statistical analysis

We performed the Spearman’s rank correlation analysis to test the association between a ranked variable and a measurement variable (e.g., the change in the relative abundance of an OTU along an identified progression path), and the Wilcoxon rank-sum test to evaluate the difference of the microbial compositions between two groups of samples. We performed the *χ*^2^ test to explore the dependence between two sets of categorical variables. If necessary, *p*-values were adjusted by the DS-FDR method [39] for multiple testing correction. We performed the ANOVA analysis to compare the alpha diversities of the identified clusters.

## Supporting information

S1 Fig Removing samples containing less than 10^4^ reads. A total of 37 samples were excluded from downstream analysis.
(PDF)

S2 Fig Identifying disease-related microorganisms using the LOGO algorithm. (**a**)The regularization parameter λ was estimated through ten-fold cross-validation. (**b**) By using a cutoff of 0.001, a total of 172 OTUs were identified to be related to disease development.
(PDF)

S3 Fig Estimating regularization parameter λ and kernel width *σ* of the DDRTree algorithm using the elbow method. The optimal *σ* and λ were estimated to be 0.5 and 150, respectively.
(PDF)

S4 Fig Comparison of inflammation activities of patients with the same CD behaviors in Cluster 4 and Cluster 5. Active inflammation was measured by fecal calprotectin >150 *μ*g/g.
(PDF)

S5 Fig OTUs with significant changes in relative abundance along at least one progression path. (PDF)

S6 Fig Spearman’s rank correlation analysis of selected OTUs for which the relative abundances were significantly decreased along the four modeled progression paths. (PDF)

S7 Fig Spearman’s rank correlation analysis of selected OTUs for which the relative abundances were significantly increased along the four modeled progression paths. (PDF)

S8 Fig Toy example illustrating how to use static samples to form pseudo-time series data. Each point presents a sample, and the solid line represents the identified progression paths. The static samples were projected onto the identified progression paths. Here, the projection of a sample was defined as a point on a progression path that is closest to the sample. By using the healthy controls as the baseline, the static samples were ordered along a path according to the extent to which the disease progressed from an inflammatory phenotype toward intestinal stricture and penetration. The ordered samples can be viewed as pseudo-time series data.
(PDF)

S9 Fig Microbial interaction networks inferred by the gLV method applied to pseudo-time series data recovered from modeled disease progression paths. Each node represents an OTU, its size is proportional to the number of edges directed out of the node (i.e., out-degree), and its face color represents the sign of the correlation of the relative abundance of the OTU with a progression path (red: positive, blue: negative).
(PDF)

S10 Fig Heatmap of KEGG pathways that were significantly disrupted along at least one of the modeled disease progression paths. Each row represents a pathway, and each column represents a patient sample. The samples were first ordered by cluster labels and then by progression distances. For the purpose of visualization, the pathway activity was log-transformed and scaled into the range of [0, 1].
(PDF)

S11 Fig KEGG pathways that were significantly disrupted along at least one disease progression path. (PDF)

S1 Table Summary of the study cohort used in the analysis. (PDF)

S2 Table Detailed clinical information of the study cohort used in the analysis. (XLSX)

S3 Table Pairwise comparisons of alpha diversities of identified clusters. The level of significance was assessed by ANOVA. ns: not significant.
(PDF)

S4 Table KEGG pathways that were significantly disrupted along at least one of the modeled progression paths. (XLSX)
