# Supplementary material for: Computational approach to modeling microbiome landscapes associated with chronic human disease progression
Source: PLoS Comput Biol. 2022 Aug 4;18(8):e1010373. doi: 10.1371/journal.pcbi.1010373 (PMC9380910; doi:10.1371/journal.pcbi.1010373)

S1 Fig. Removing samples containing less than 10^4 reads. A total of 37 samples were excluded from downstream analysis.

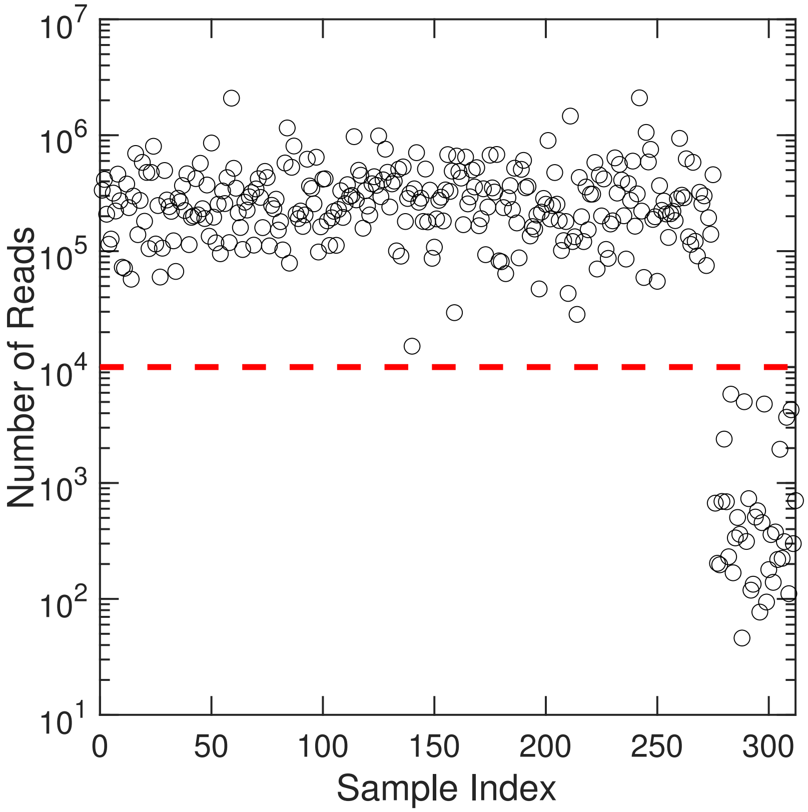

Supplement: S1 Fig — A total of 37 samples were excluded from downstream analysis. (PDF) [file pcbi.1010373.s001.pdf]
