# Supplementary material for: Computational approach to modeling microbiome landscapes associated with chronic human disease progression
Source: PLoS Comput Biol. 2022 Aug 4;18(8):e1010373. doi: 10.1371/journal.pcbi.1010373 (PMC9380910; doi:10.1371/journal.pcbi.1010373)
Supplement: S2 Fig — (a)The regularization parameter λ was estimated through ten-fold cross-validation. (b) By using a cutoff of 0.001, a total of 172 OTUs were identified to be related to disease development. (PDF) [file pcbi.1010373.s002.pdf]

S2 Fig. Identifying disease-related microorganisms using the LOGO algorithm. (**a**)The regularization parameter λ was estimated through ten-fold cross-validation. (**b**) By using a cutoff of 0.001, a total of 172 OTUs were identified to be related to disease development.

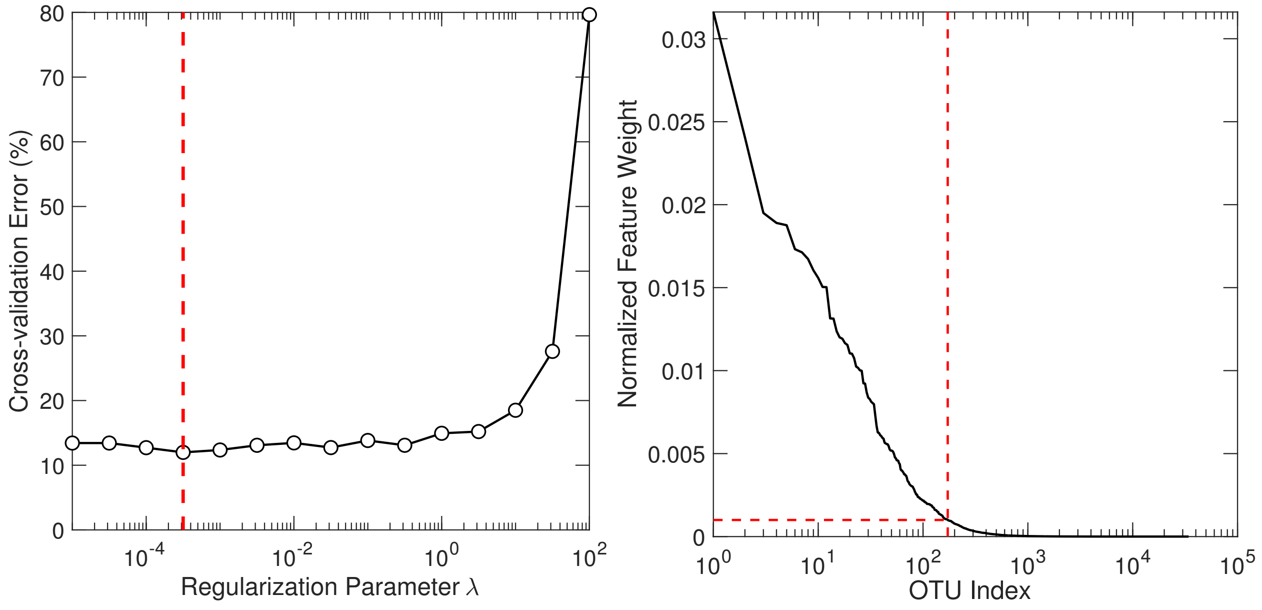

(a) **(b)**
