# Supplementary material for: Computational approach to modeling microbiome landscapes associated with chronic human disease progression
Source: PLoS Comput Biol. 2022 Aug 4;18(8):e1010373. doi: 10.1371/journal.pcbi.1010373 (PMC9380910; doi:10.1371/journal.pcbi.1010373)
Supplement: S3 Fig — The optimal σ and λ were estimated to be 0.5 and 150, respectively. (PDF) [file pcbi.1010373.s003.pdf]

S3 Fig. Estimating regularization parameter λ and kernel width σ of the DDRTree algorithm using the elbow method. The optimal σ and λ were estimated to be 0.5 and 150, respectively.

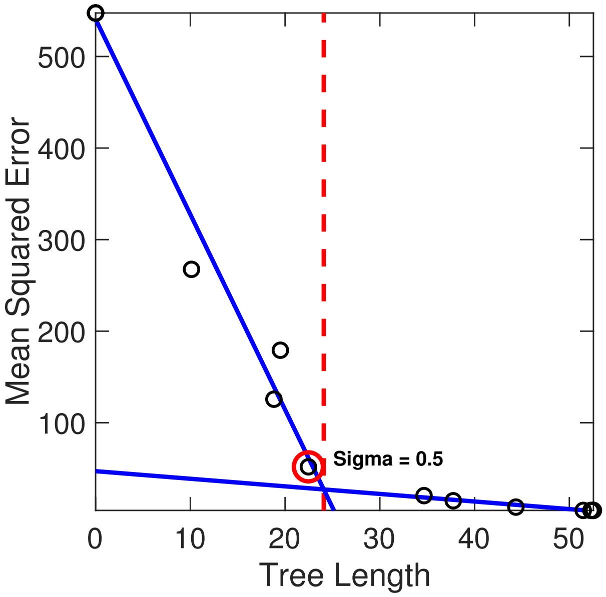

(a)

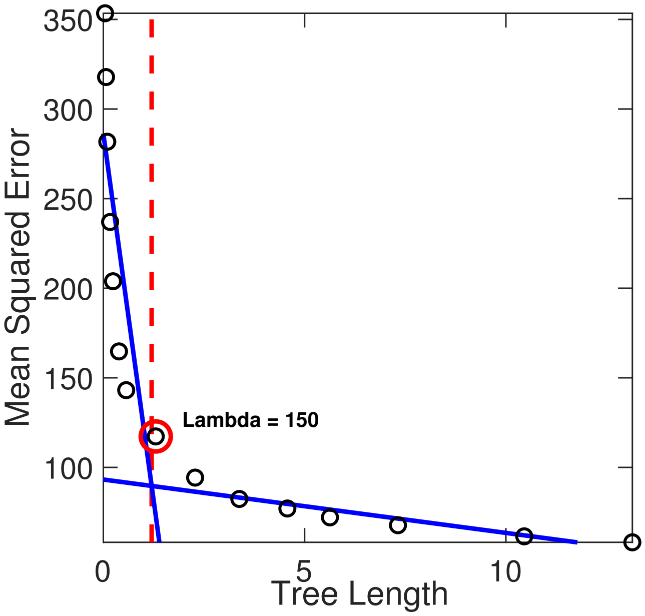

(b)
