# Supplementary material for: Computational approach to modeling microbiome landscapes associated with chronic human disease progression
Source: PLoS Comput Biol. 2022 Aug 4;18(8):e1010373. doi: 10.1371/journal.pcbi.1010373 (PMC9380910; doi:10.1371/journal.pcbi.1010373)

S4 Fig. Comparison of inflammation activities of patients with the same CD behaviors in Cluster 4 and Cluster 5. Active inflammation was measured by fecal calprotectin >150 μg/g.

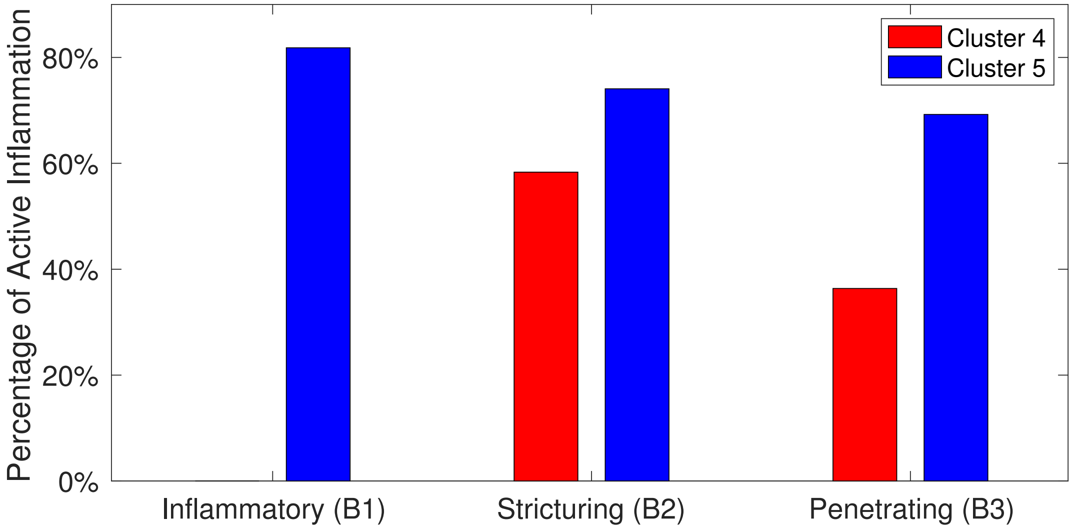

Supplement: S4 Fig — Active inflammation was measured by fecal calprotectin >150 μg/g. (PDF) [file pcbi.1010373.s004.pdf]
