# Supplementary material for: Computational approach to modeling microbiome landscapes associated with chronic human disease progression
Source: PLoS Comput Biol. 2022 Aug 4;18(8):e1010373. doi: 10.1371/journal.pcbi.1010373 (PMC9380910; doi:10.1371/journal.pcbi.1010373)

S5 Fig. OTUs with significant changes in relative abundance along at least one progression path.

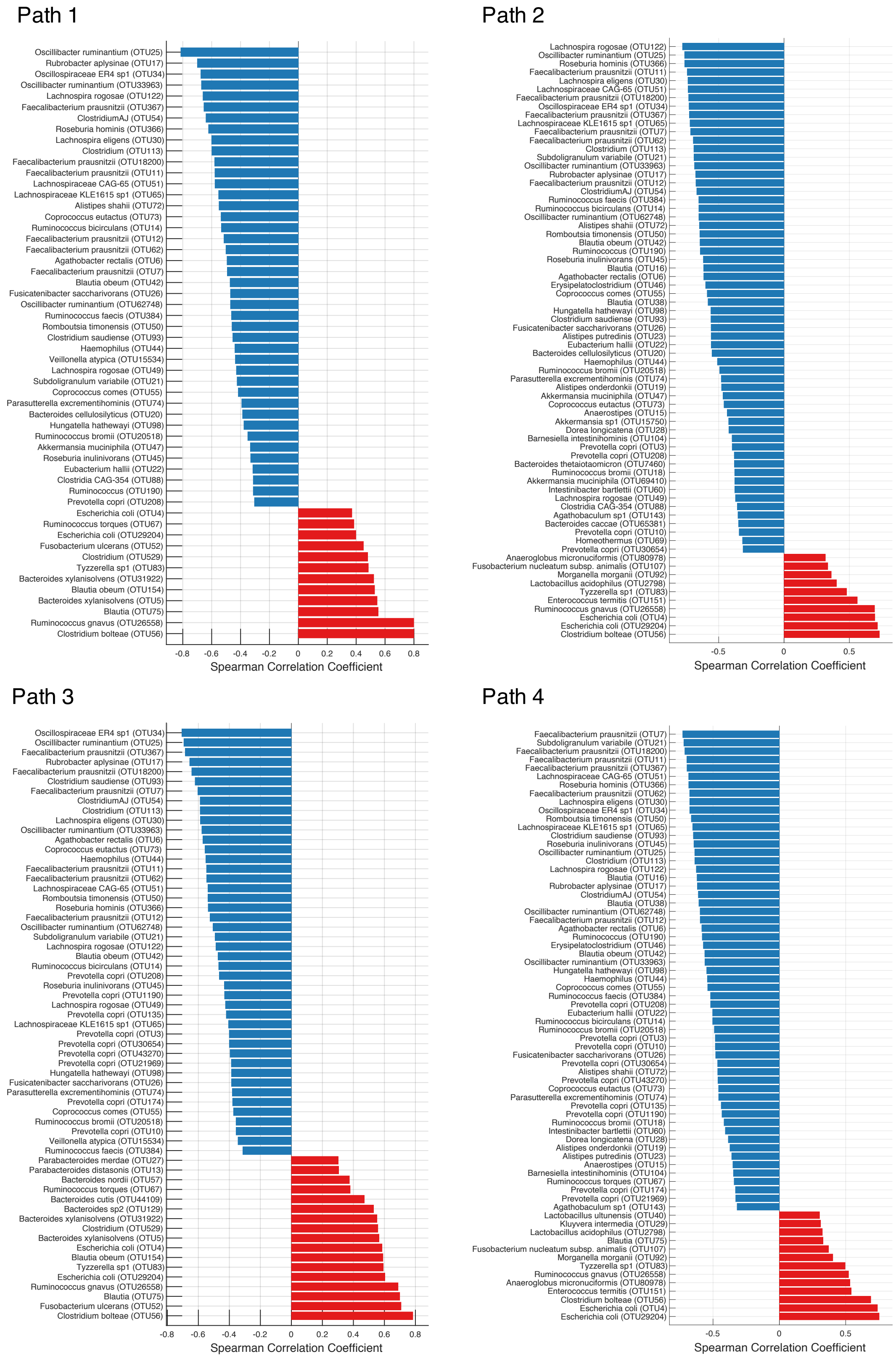

Supplement: S5 Fig — (PDF) [file pcbi.1010373.s005.pdf]
