# Supplementary material for: Computational approach to modeling microbiome landscapes associated with chronic human disease progression
Source: PLoS Comput Biol. 2022 Aug 4;18(8):e1010373. doi: 10.1371/journal.pcbi.1010373 (PMC9380910; doi:10.1371/journal.pcbi.1010373)

S7 Fig. Spearman's rank correlation analysis of selected OTUs for which the relative abundances were significantly increased along the four modeled progression paths.

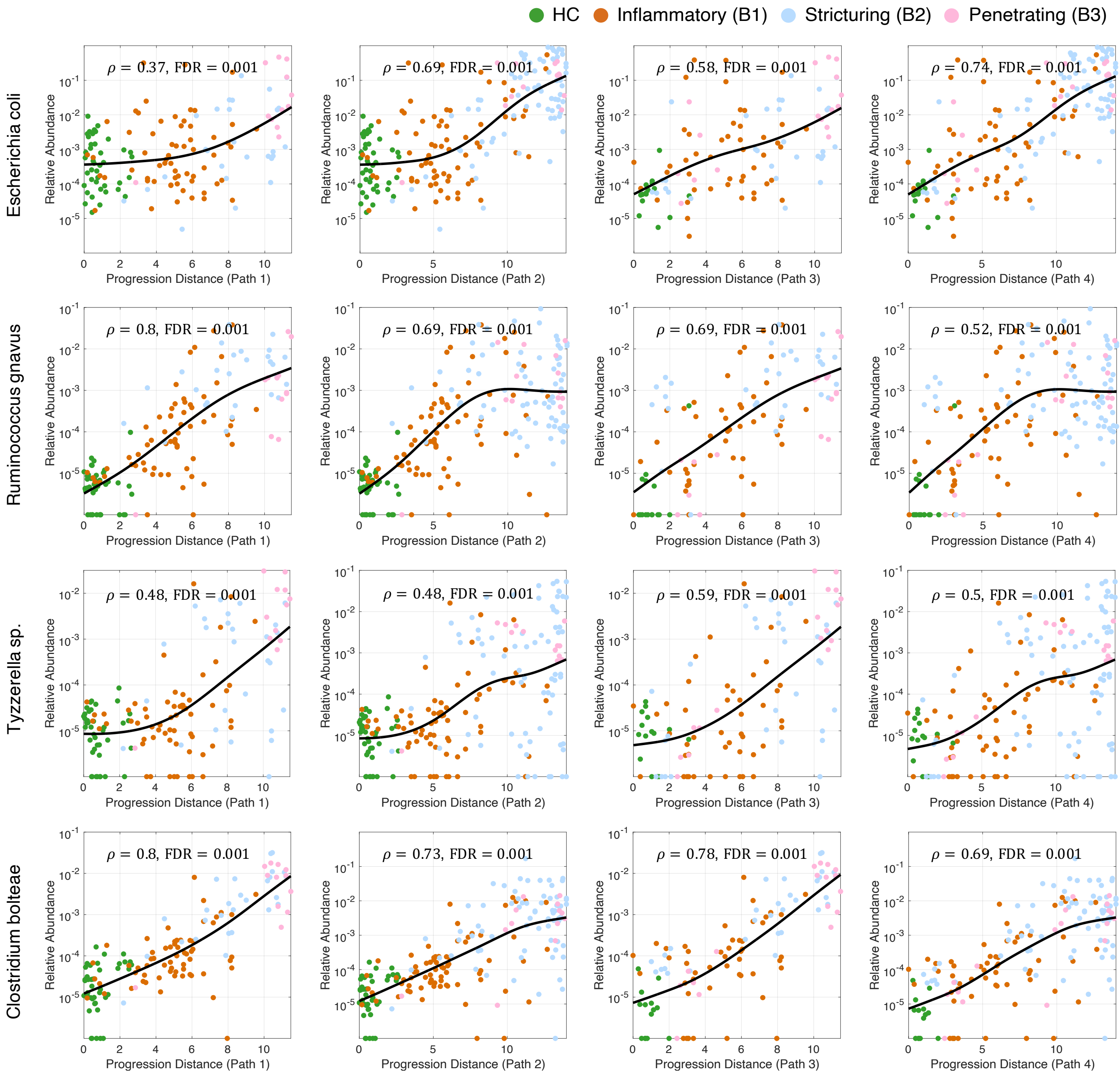

Supplement: S7 Fig — (PDF) [file pcbi.1010373.s007.pdf]
