# Supplementary material for: Computational approach to modeling microbiome landscapes associated with chronic human disease progression
Source: PLoS Comput Biol. 2022 Aug 4;18(8):e1010373. doi: 10.1371/journal.pcbi.1010373 (PMC9380910; doi:10.1371/journal.pcbi.1010373)

S8 Fig. Toy example illustrating how to use static samples to form pseudo-time series data. Each point presents a sample, and the solid line represents the identified progression paths. The static samples were projected onto the identified progression paths. Here, the projection of a sample was defined as a point on a progression path that is closest to the sample. By using the healthy controls as the baseline, the static samples were ordered along a path according to the extent to which the disease progressed from an inflammatory phenotype toward intestinal stricture and penetration. The ordered samples can be viewed as pseudo-time series data.

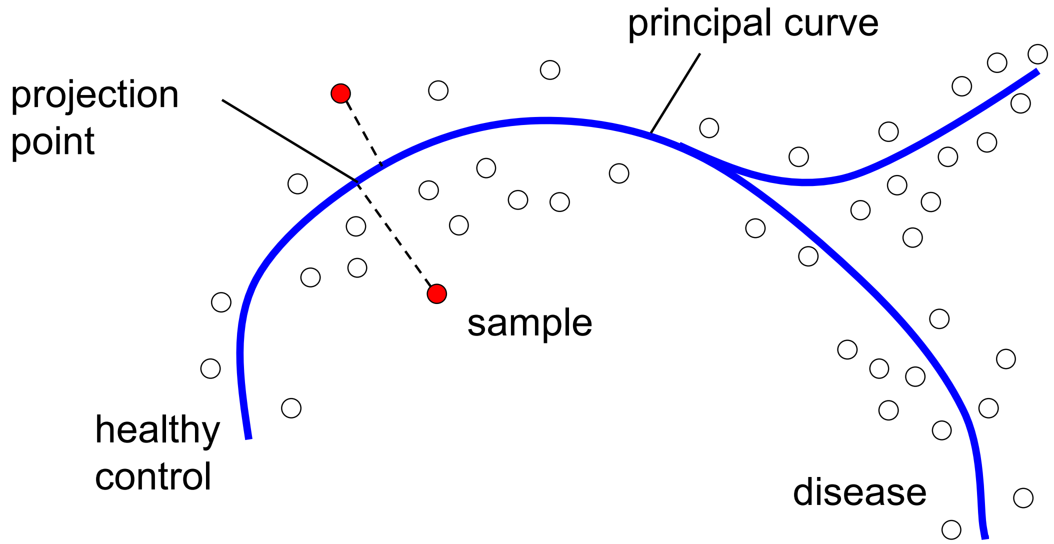

Supplement: S8 Fig — Each point presents a sample, and the solid line represents the identified progression paths. The static samples were projected onto the identified progression paths. Here, the projection of a sample was defined as a point on a progression path that is closest to the sample. By using the healthy controls as the baseline, the static samples were ordered along a path according to the extent to which the disease progressed from an inflammatory phenotype toward intestinal stricture and penetration. The ordered samples can be viewed as pseudo-time series data. (PDF) [file pcbi.1010373.s008.pdf]
