# Supplementary material for: Computational approach to modeling microbiome landscapes associated with chronic human disease progression
Source: PLoS Comput Biol. 2022 Aug 4;18(8):e1010373. doi: 10.1371/journal.pcbi.1010373 (PMC9380910; doi:10.1371/journal.pcbi.1010373)
Supplement: S9 Fig — Each node represents an OTU, its size is proportional to the number of edges directed out of the node (i.e., out-degree), and its face color represents the sign of the correlation of the relative abundance of the OTU with a progression path (red: positive, blue: negative). (PDF) [file pcbi.1010373.s009.pdf]

S9 Fig. Microbial interaction networks inferred by the gLV method applied to pseudo-time series data recovered from modeled disease progression paths. Each node represents an OTU, its size is proportional to the number of edges directed out of the node (i.e., out-degree), and its face color represents the sign of the correlation of the relative abundance of the OTU with a progression path (red: positive, blue: negative).

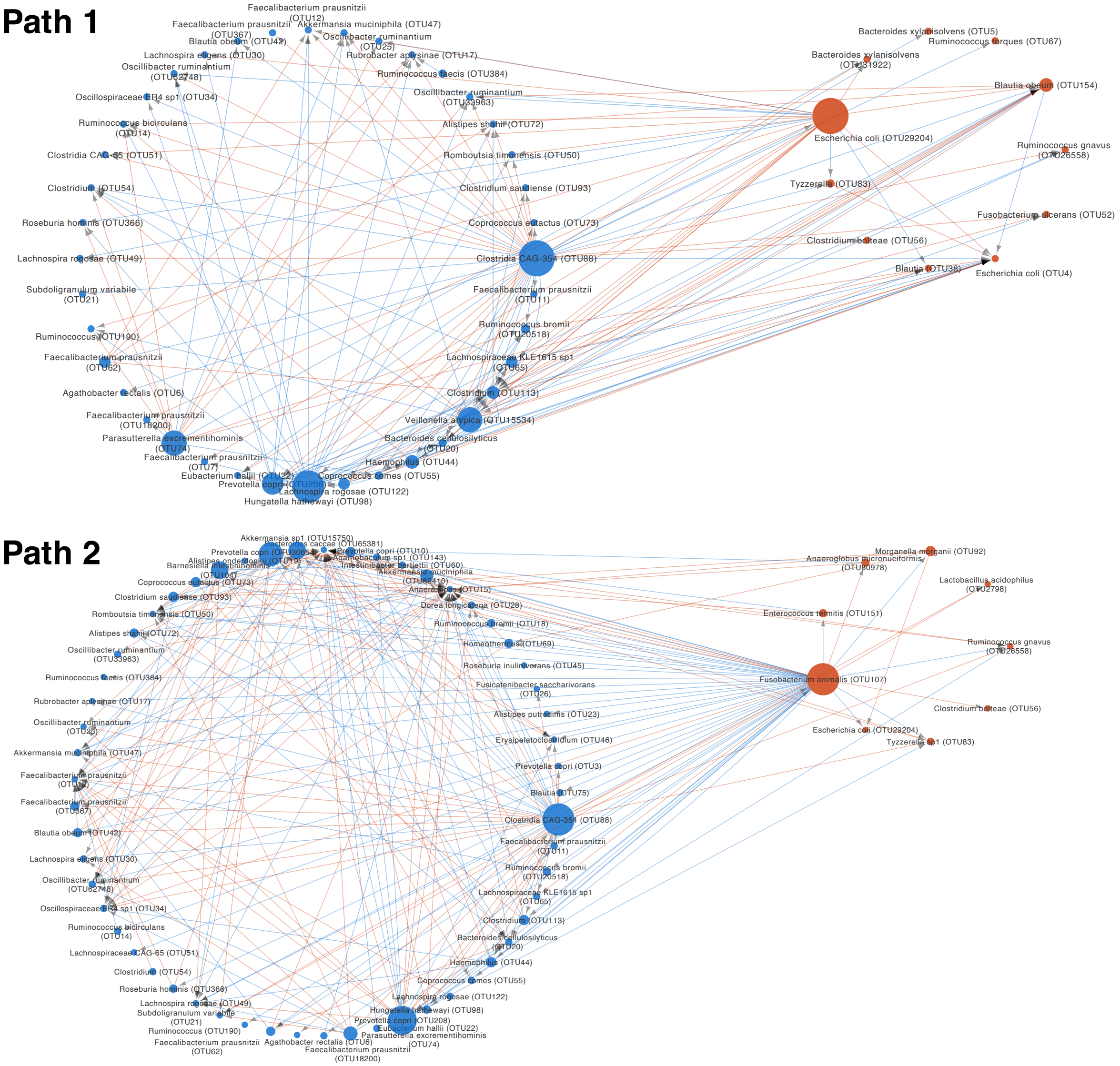

Path 3

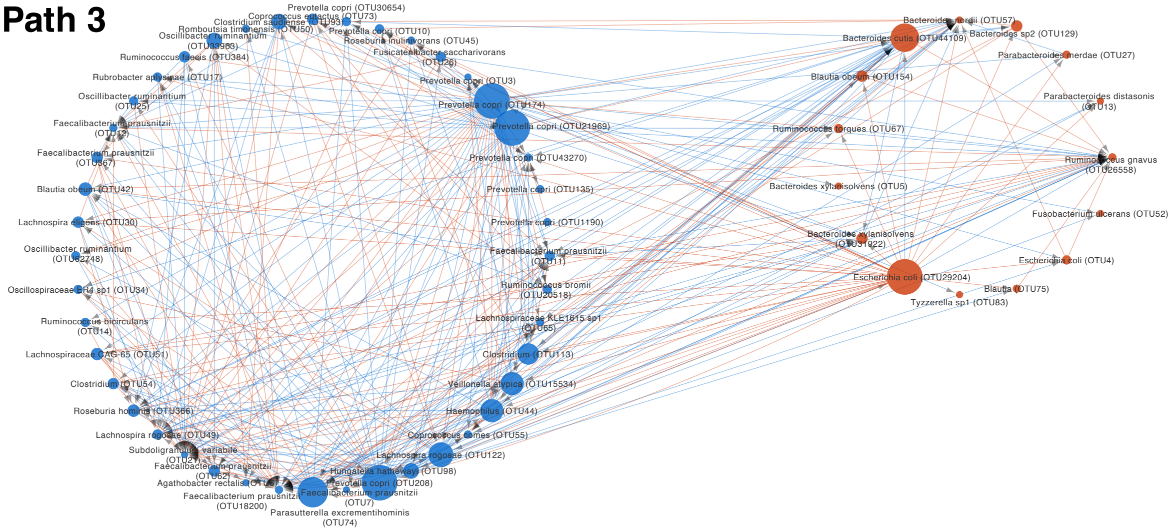

Prevotella copri (OTU30654)
Coprococcus eutactus (OTU73)
Clostridium saudiense (OTU93)
Romboutsia timonensis (OTU50)
Prevotella copri (OTU10)
Oscillibacter ruminantium (OTU33963)
Roseburia inulinivorans (OTU45)
Fusicatenibacter saccharivorans (OTU26)
Ruminococcus faecis (OTU384)
Rubrobacter aplysinae (OTU17)
Prevotella copri (OTU3)
Oscillibacter ruminantium (OTU25)
Prevotella copri (OTU174)
Faecalibacterium prausnitzii (OTU12)
Prevotella copri (OTU21969)
Faecalibacterium prausnitzii (OTU367)
Prevotella copri (OTU43270)
Blautia obeum (OTU42)
Prevotella copri (OTU135)
Lachnospira eligens (OTU30)
Prevotella copri (OTU1190)
Oscillibacter ruminantium (OTU62748)
Faecalibacterium prausnitzii (OTU11)
Oscillospiraceae ER4 sp1 (OTU34)
Ruminococcus bromii (OTU20518)
Ruminococcus bicirculans (OTU14)
Lachnospiraceae KLE1615 sp1 (OTU65)
Lachnospiraceae CAG-65 (OTU51)
Clostridium (OTU113)
Clostridium (OTU54)
Veillonella atypica (OTU15534)
Roseburia hominis (OTU366)
Haemophilus (OTU44)
Lachnospira rogosae (OTU49)
Coprococcus comes (OTU55)
Subdoligranulum variabile (OTU21)
Lachnospira rogosae (OTU122)
Faecalibacterium prausnitzii (OTU62)
Hungatella hathewayi (OTU98)
Agathobacter rectalis (OTU6)
Prevotella copri (OTU208)
Faecalibacterium prausnitzii (OTU18200)
Faecalibacterium prausnitzii (OTU7)
Parasutterella excrementihominis (OTU74)
Bacteroides fordii (OTU57)
Bacteroides cutis (OTU44109)
Bacteroides sp2 (OTU129)
Parabacteroides merdae (OTU27)
Blautia obeum (OTU154)
Parabacteroides distasonis (OTU13)
Ruminococcus torques (OTU67)
Ruminococcus gnavus (OTU26558)
Bacteroides xylanisolvens (OTU5)
Fusobacterium ulcerans (OTU52)
Bacteroides xylanisolvens (OTU31022)
Escherichia coli (OTU4)
Escherichia coli (OTU29204)
Blautia (OTU75)
Tyzzerella sp1 (OTU83)

Path 4

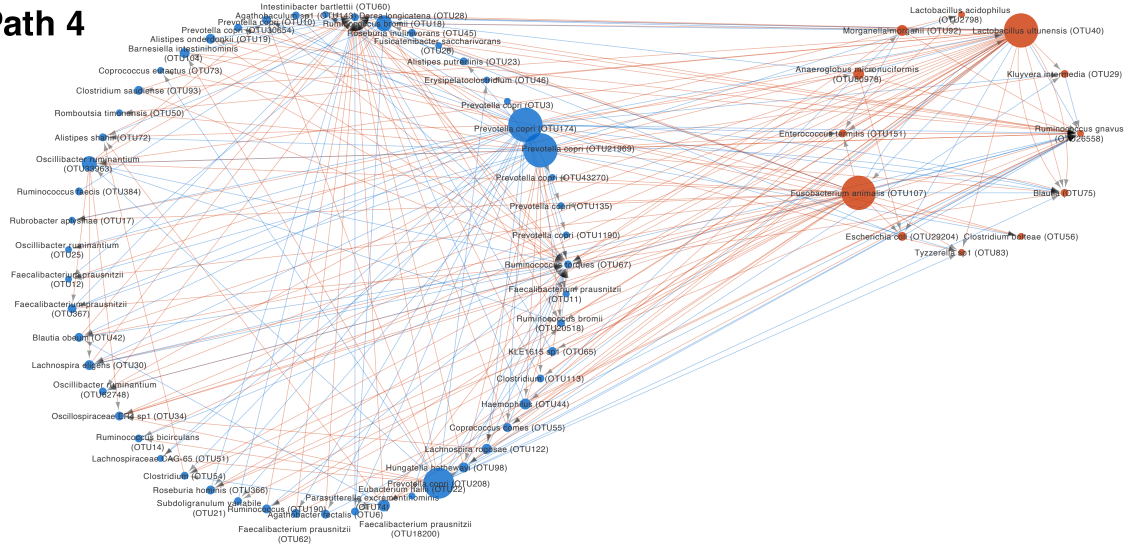

Intestinibacter bartlettii (OTU60)
Agathobaculum sp1 (OTU143)
Dorea longicatena (OTU28)
Prevotella copri (OTU10)
Ruminococcus bromii (OTU18)
Prevotella copri (OTU30654)
Roseburia inulinivorans (OTU45)
Alistipes onderdonkii (OTU19)
Fusicatenibacter saccharivorans (OTU26)
Barnesiella intestinihominis (OTU104)
Alistipes putredinis (OTU23)
Coprococcus eutactus (OTU73)
Erysipelatoclostridium (OTU46)
Clostridium saudiense (OTU93)
Prevotella copri (OTU3)
Romboutsia timonensis (OTU50)
Prevotella copri (OTU174)
Alistipes shahii (OTU72)
Prevotella copri (OTU21969)
Oscillibacter ruminantium (OTU33963)
Prevotella copri (OTU43270)
Ruminococcus faecis (OTU384)
Prevotella copri (OTU135)
Rubrobacter aplysinae (OTU17)
Prevotella copri (OTU1190)
Oscillibacter ruminantium (OTU25)
Ruminococcus torques (OTU67)
Faecalibacterium prausnitzii (OTU12)
Faecalibacterium prausnitzii (OTU11)
Faecalibacterium prausnitzii (OTU367)
Ruminococcus bromii (OTU20518)
Blautia obeum (OTU42)
KLE1615 sp1 (OTU65)
Lachnospira eligens (OTU30)
Clostridium (OTU113)
Oscillibacter ruminantium (OTU62748)
Haemophilus (OTU44)
Oscillospiraceae ER4 sp1 (OTU34)
Coprococcus comes (OTU55)
Ruminococcus bicirculans (OTU14)
Lachnospira rogosae (OTU122)
Lachnospiraceae CAG-65 (OTU51)
Hungatella hathewayi (OTU98)
Clostridium (OTU54)
Prevotella copri (OTU208)
Roseburia hominis (OTU366)
Eubacterium hallii (OTU22)
Subdoligranulum variabile (OTU21)
Parasutterella excrementihominis (OTU74)
Ruminococcus (OTU190)
Agathobacter rectalis (OTU6)
Faecalibacterium prausnitzii (OTU62)
Faecalibacterium prausnitzii (OTU18200)
Lactobacillus acidophilus (OTU2798)
Morganella morganii (OTU92)
Lactobacillus ultunensis (OTU40)
Anaeroglobus micronuciformis (OTU80978)
Kluyvera intermedia (OTU29)
Enterococcus hirae (OTU151)
Ruminococcus gnavus (OTU26558)
Fusobacterium animalis (OTU107)
Blautia (OTU75)
Escherichia coli (OTU29204)
Clostridium bolteae (OTU56)
Tyzzerella sp1 (OTU83)
