# Supplementary material for: Computational approach to modeling microbiome landscapes associated with chronic human disease progression
Source: PLoS Comput Biol. 2022 Aug 4;18(8):e1010373. doi: 10.1371/journal.pcbi.1010373 (PMC9380910; doi:10.1371/journal.pcbi.1010373)

S10 Fig. Heatmap of KEGG pathways that were significantly disrupted along at least one of the modeled disease progression paths. Each row represents a pathway, and each column represents a patient sample. The samples were first ordered by cluster labels and then by progression distances. For the purpose of visualization, the pathway activity was log-transformed and scaled into the range of [0, 1].

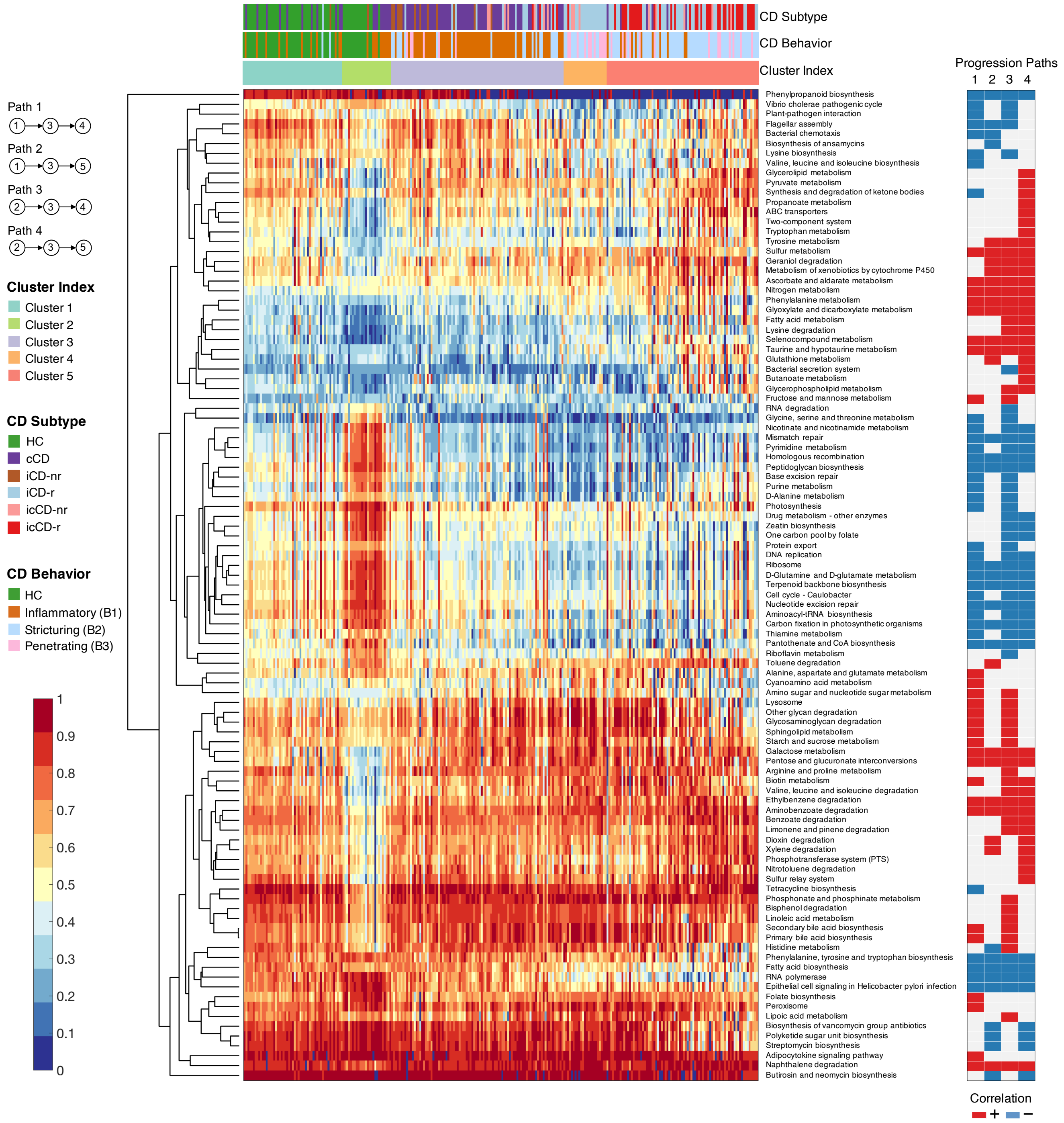

Supplement: S10 Fig — Each row represents a pathway, and each column represents a patient sample. The samples were first ordered by cluster labels and then by progression distances. For the purpose of visualization, the pathway activity was log-transformed and scaled into the range of [0, 1]. (PDF) [file pcbi.1010373.s010.pdf]
