# Supplementary material for: Computational approach to modeling microbiome landscapes associated with chronic human disease progression
Source: PLoS Comput Biol. 2022 Aug 4;18(8):e1010373. doi: 10.1371/journal.pcbi.1010373 (PMC9380910; doi:10.1371/journal.pcbi.1010373)
Supplement: S1 Table — (PDF) [file pcbi.1010373.s012.pdf]

S1 Table. Summary of the study cohort used in the analysis.

**Clinical Characteristics**	**HC** ($n = 9$)	**cCD** ($n = 18$)	**iCD-nr** ($n = 2$)	**iCD-r** ($n = 15$)	**icCD-nr** ($n = 4$)	**icCD-r** ($n = 10$)
Male/Female	3/6	8/10	0/2	9/6	2/2	4/6
Mean BMI<br>(lower 95% - upper 95%)	23.6<br>(22.0 - 25.1)	25.1<br>(23.4 - 26.7)	29<br>(21.2 - 36.8)	26<br>(23.6 - 28.4)	27.0<br>(23.0 - 31.0)	23.4<br>(20.5 - 26.4)
Mean duration of years<br>(lower 95% - upper 95%)	/<br>/	14.5<br>(7.8 - 21.2)	26.5<br>(11.8 - 41.2)	19.5<br>(8.1 - 30.9)	15.3<br>(0.2 - 30.3)	24.2<br>(17.0 - 31.4)
Disease location						
L1 Ileal (%)	/	0 (0)	2 (100)	14 (93)	0 (0)	0 (0)
L2 Colonic (%)	/	18 (100)	0 (0)	0 (0)	0 (0)	0 (0)
L3 Ileocolonic (%)	/	0 (0)	0 (0)	0 (0)	3 (75)	8 (80)
L1+L4 Ileal and Upper-GI (%)	/	0 (0)	0 (0)	1 (7)	0 (0)	0 (0)
L3+L4 Ileocolonic and Upper-GI (%)	/	0 (0)	0 (0)	0 (0)	1 (25)	2 (20)
Crohn's disease behavior						
B1 Inflammatory (%)	/	16 (88.8)	1 (50)	1 (7)	3 (75)	1 (10)
B2 Stricturing (%)	/	1 (5.6)	1 (50)	12 (80)	1 (25)	5 (50)
B3 Penetrating (%)	/	1 (5.6)	0 (0)	2 (13)	0 (0)	4 (40)

HC: healthy control; cCD: colonic Crohn's disease; iCD: ileal Crohn's disease; icCD: ileocolonic Crohn's disease; -r/nr: with/without ileocaecal resection
