# Supplementary material for: Computational approach to modeling microbiome landscapes associated with chronic human disease progression
Source: PLoS Comput Biol. 2022 Aug 4;18(8):e1010373. doi: 10.1371/journal.pcbi.1010373 (PMC9380910; doi:10.1371/journal.pcbi.1010373)
Supplement: S3 Table — The level of significance was assessed by ANOVA. ns: not significant. (PDF) [file pcbi.1010373.s014.pdf]

S3 Table. Pairwise comparisons of alpha diversities of identified clusters. The level of significance was assessed by ANOVA. ns: not significant.

**Comparison**	**Chao 1 Index**		**Shannon Index**	
	Mean Difference	*p*-value	Mean Difference	*p*-value
Cluster 1 vs. Cluster 2	681.8	<0.001	1.2	< 0.001
Cluster 1 vs. Cluster 3	513.9	<0.001	0.8	< 0.001
Cluster 1 vs. Cluster 4	1353.6	<0.001	2.0	<0.001
Cluster 1 vs. Cluster 5	1550.7	<0.001	2.5	<0.001
Cluster 2 vs. Cluster 3	ns	ns	ns	ns
Cluster 2 vs. Cluster 4	671.9	<0.001	0.8	0.03
Cluster 2 vs. Cluster 5	869.0	<0.001	1.3	<0.001
Cluster 3 vs. Cluster 4	839.7	<0.001	1.2	<0.001
Cluster 3 vs. Cluster 5	1036.8	<0.001	1.8	<0.001
Cluster 4 vs. Cluster 5	ns	ns	ns	ns
